# InSitu Integrated Fabrication for Multi‐Interface Stabilized and Highly Durable Polyaniline@Graphene Oxide/Polyether Ether Ketone Special Separation Membranes

**DOI:** 10.1002/advs.202302654

**Published:** 2023-06-28

**Authors:** Ziyu Lin, Jundong Zhong, Runyin Sun, Yingzhen Wei, Zhonghui Sun, Wenying Li, Liyuan Chen, Yirong Sun, Haibo Zhang, Jinhui Pang, Zhenhua Jiang

**Affiliations:** ^1^ Key Laboratory of High Performance Plastics (Jilin University) Ministry of Education National & Local Joint Engineering Laboratory for Synthetic Technology of High Performance Polymer College of Chemistry Jilin University Changchun 130012 P. R. China

**Keywords:** confined polymerization, polyaniline integrated growth, polyether ether ketone porous membranes, special separation membranes, stable graphene oxide layers

## Abstract

Special separation membranes are widely employed for separation and purification purposes under challenging operating conditions due to their low energy consumption, excellent solvent, and corrosion resistance. However, the development of membranes is limited by corrosion‐resistant polymer substrates and precise interfacial separation layers. Herein, polyaniline (PANI) is employed to achieve insitu anchoring of multiple interfaces, resulting in the fabrication of polyaniline@graphene oxide/polyether ether ketone (PANI@GO/PEEK) membranes. Insitu growth of PANI achieves the adequate bonding of the PEEK substrate and GO separation interface, which solves the problem of solution processing of PEEK and the instability of GO layers. By bottom‐up confined polymerization of aniline, it could control the pore size of the separation layer, correct defects, and anchor among polymer, nano‐separation layer, and nano‐sheet. The mechanism of membrane construction within the confined domain and micro‐nano structure modulation is further explored. The membranes demonstrate exceptional stability realizing over 90% rejection in 2 m HCl, NaOH, and high temperatures. Additionally, –membranes exhibit remarkable durability after 240 days immersion and 100 h long‐term operation, which display the methanol flux of 50.2 L m^−2^ h^−1^ and 92% rejection of AF (585 g mol^−1^). This method substantially contributes to special separation membranes by offering a novel strategy.

## Graphical abstract

Multiple interfaces are stabilized by growing polyaniline to create polyaniline@graphene oxide/polyether ether ketone membranes in an insitu integrated manner, which also realizes the conversion of solvent‐resistant polyether ether ketone (PEEK) substrates. The membrane achieves pore size regulation of the separation layer, repairing defect and anchoring among the PEEK substrate, the nano‐separation layer, and the nano‐sheet layer to obtain highly stable and durable special separation membranes.

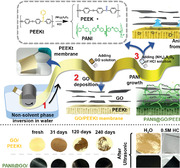

## 1 Introduction

Energy and environmental issues have emerged as urgent challenges for humanity problems with society striving for low‐carbon, environmentally friendly, and highly efficient development. At present, an area of particular concern is the separation of chemicals, which accounts for ≈10–15% of the world's total energy consumption.^[^ ^1^, ^2^ ^]^ The purification of organic solvents to prepare these chemicals and pharmaceuticals involves a large number of chemical separations. Unfortunately, the current separation processes rely on energy‐intensive distillation separation technologies, contributing to more than 80% of the total energy consumption of chemical separations.^[^ ^3^, ^4^ ^]^ To address these challenges, membrane separation technologies have attracted more attention in industrial applications due to their unique advantages such as high efficiency, low cost, and sustainability.^[^ ^4^, ^5^ ^]^ Consequently, membrane‐based separation technologies hold immense potential in chemical separation processes and are expected to revolutionize the handling of organic chemical feedstock and organic solvent handling processes.^[^ ^6^, ^7^ ^]^ However, the ability of membrane separation processes to operate under extreme conditions, such as highly acidic or alkaline solutions, limits the use of membrane separation processes. Such conditions pose rigorous tests for membrane materials regarding their separation capabilities.

In the separation and purification process, special separation membranes garnered considerable attention due to their promising application prospects in solvent and corrosion resistance. Although three fields of organic solvent separation membranes (organic solvent nanofiltration [OSN], organic solvent reverse osmosis [OSRO], and organic solvent forward osmosis [OSFO]) have been proposed in the early 1970s.^[^ ^2^, ^4^ ^]^ But so far, despite their inception several decades ago, there are almost no large‐scale applications. The primary obstacle lies in the challenges associated with developing membrane materials with stability and high selectivity. The rapid development of polymers provides a plethora of excellent materials for the preparation of special separation membranes.^[^ ^8^, ^9^ ^]^ Notably, various polymer resins, such as polyimide, polyaryl ether ketone (PAEK), polybenzimidazole, poly siloxane, and polytetra fluoro ethylene, have been reported as suitable candidates for organic solvent separation membranes.^[^ ^10^, ^11^ ^]^ Among them, PAEK series polymers are known for solvent resistance. Polyether ether ketone (PEEK), a typical representative of PAEK, has become an area of interest for the preparation of separation membrane materials because of its outstanding solvent resistance. However, PEEK has a problem limiting its use in the field of separation membranes due to the difficulty in solution processing and interfacial incompatibility.^[^ ^12^ ^]^ Currently, the process of preparing PEEK membranes involves the initial preparation of amorphous PAEK polymer (PEEKt), followed by the production of separation precursor membranes by the nonsolvent induced phase separation (NIPS) method. Finally, these precursor membranes are combined with nonhomogeneous hydrolysis ways to produce PEEK separation membranes.^[^ ^13^, ^14^ ^]^

The challenge of developing highly permeable and selective membrane materials for organic solvent separation is the construction of efficient separation interfaces besides the solvent stability of polymeric substratemembranes. To address this challenge, two–dimensional (2D) membrane materials have emerged as a prominent area of research in the field of separation. These materials offer regular layered pore channels, rich interfacial structures, and modifiable fluid transport paths in nanochannels, which provides a means to regulate the fabrication of pore channels with specific selectivity for OSN.^[^ ^4^, ^15^ ^]^ Compared with conventional separation membranes, graphene oxide (GO) membranes have gained increasing attention due to their unique properties.^[^ ^16^, ^17^ ^]^ As 2D material, GO is characterized by oxygen‐containing functional groups and non‐oxidized sp^2^ structural domains. The non‐oxidized region between GO sheets creates a frictionless nanochannel that allows ultrafast transport of water molecules.^[^ ^18^ ^]^ The tunable nanochannel can be a selective barrier to all hydrated radii more significantly than the nanochannel. However, incomplete stacking of GO sheets can lead to microporous defects (framework defects) between the layers, which can disrupt the microstructure of GO.^[^ ^19^, ^20^ ^]^ Meanwhile, due to extensive oxygen‐containing functional groups on the surface of GO, the problem of GO sheets swelling and membrane rupture during long‐time separation becomes a bottleneck for development. The durability of GO membranes under extreme conditions also restricts their practical application. Consequently, it is imperative to control the stacking of GO nanosheets to form suitable interlayer nanochannels, considering the stability of membrane while keeping the desired separation efficacy.^[^ ^6^, ^16^, ^19^ ^]^ The GO‐based membranes are improved by means of nanoparticles, nanofibers, polymer electrolytes, charges, and covalent cross‐linking, to achieve the desired pore structure, surface functionality, and separation performance.^[^ ^4^, ^18^, ^19^, ^20^, ^21^, ^22^, ^23^ ^]^ These efforts have improved membrane stability while simultaneously enhancing the membrane efficiency and separation, among which covalent cross‐linking and polymer networks anchoring the 2D sheets are advantageous in improving membrane stability.^[^ ^21^, ^23^, ^24^ ^]^ But GO is usually not available on its own due to its high rigidity and high cost. As a result, commercially available polymeric porous membranes are frequently employed as substrate materials for these membranes. Previous studies have primarily focused on constructing membrane materials by vacuum‐assisted filtration or post‐substrate modification, which may suffer from problems such as high cost, interface shedding, and breakage.^[^ ^24^, ^25^ ^]^

**Figure 1:**
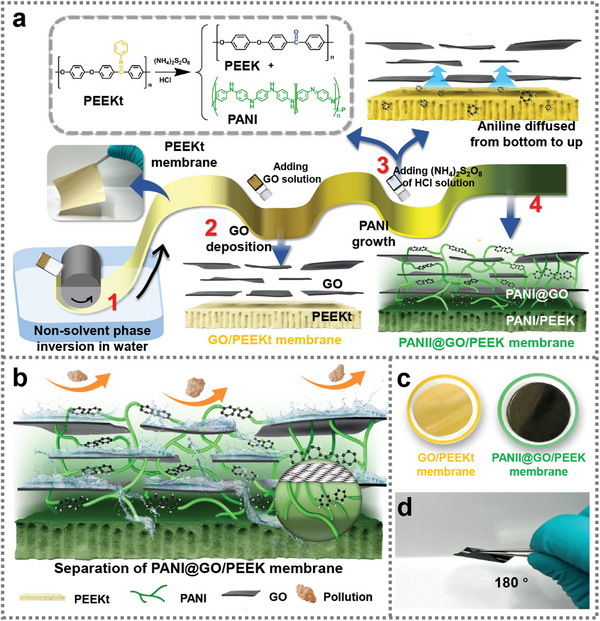
a) Schematic illustration of the formation method and microstructure of the PANI@GO/PEEK composite membrane (inset is PEEKt oxidation to synthesize PANI/PEEK composites). b) The separation progress of the PANI@GO/PEEK membrane. c,d) The physical images of the GO/PEEKt membrane and PANI@GO/PEEK membrane.

Herein, this work presents a novel method for producing super special separation membranes by combining solvent‐resistant PEEK membranes with 2D nano‐sheet separation membranes. The key innovation lies in stabilizing multiple interfaces by growing polyaniline (PANI) in an insitu integrated manner to create polyaniline@graphene oxide/polyether ether ketone (PANI@GO/PEEK) membranes, which also achieved solution fabrication of solvent‐resistant PEEK substrate (**Figure** **1**). The integrated bottom‐up confined polymerization of aniline has anchored the multiple interfaces, which achieves pore size regulation of the separation layer and repairing defecct and anchoring among the polymer, the nano‐separation layer, and the nano‐sheet layer to obtain highly stable and efficient special separation membranes. To achieve insitu confined anchoring of multiple interfaces, aniline in the bottom layer diffused into the GO layer under the effect of HCl and (NH_4_)_2_S_2_O_8_ to realize the PANI insitu confined anchoring of multiple interfaces. The bottom‐up insitu PANI growth was created in this manner, and the distributed PANI had the positive adhesion impact on producing highly durable PANI@GO/PEEK membranes, which created the solution for the issues with GO stability and PEEK's incompatibility with the GO layer. Additionally, the process and mechanism of hydrolysis synergistic oxidative coupling polymerization of PEEKt membranes, the growth and phase behavior of PANI chains under the confined domain, and the process of micro‐nano structure modulation within the membranes were further investigated. By controlling various factors influencing the membrane formation process and pattern changes, PANI@GO/PEEK membrane exhibited super corrosion resistance and membrane stability. The proposed integrated PANI stabilization of multiple interfaces in this work holds great scientific significance in special separation and 2D membranes.

## 2 Results and Discussion

### 2.1 Characterization and Growth of the PANI@GO/PEEK Membrane

To fabricate PANI@GO/PEEK membranes, PEEKt membranes were obtained on the basis of GO vacuum‐assisted filtration deposition and PANI insitu integrated growth. PEEKt (Scheme S2, Supporting Information) was synthesized from *N*‐phenyl(4,4′‐difluorodiphenyl) ketimin (Scheme S1, Supporting Information) and 1,4‐benzenediol by nucleophilic polycondensation reaction.^[^ ^14^, ^26^ ^]^ The structures of *N*‐phenyl(4,4′‐difluorodiphenyl) ketimin (Figure S1, Supporting Information) and PEEKt (Figure S2, Supporting Information) were confirmed by ^1^H NMR. All protons of PEEKt and *N*‐phenyl(4,4′‐difluorodiphenyl) ketimin were compared precisely according to the ^1^H NMR characterization. Two distinct new peaks appear at 6.71 and 6.73 ppm, which are attributed to H_a_ on the benzene ring indicating the successful synthesis of PEEKt.^[^ ^13^, ^14^, ^26^ ^]^ Porous PEEKt membranes were obtained by the NIPS method (Figure S3, Supporting Information).^[^ ^12^ ^]^ The PEEKt membrane exhibits an obvious asymmetric microstructure (Figure S4, Supporting Information). From the cross‐section, the membrane is made up of dense layer surfaces and finger‐like pores. The membrane surface is relatively smooth and dense with the presence of micro‐nano‐pore structure. The smooth PEEKt membrane creates an ideal environment for the consistent deposition of GO and the homogenous growth of PANI.

In the prior work of our group, it was confirmed that the polymer precursor (PEEKt) can be hydrolyzed by Schiff bases to achieve the complete conversion of ketimine to ketone, which further transforms the amorphous polymer precursor into a semi‐crystalline structure.^[^ ^14^, ^27^, ^28^ ^]^ Meanwhile, excellent compatibility between PANI and PEEK can be further achieved. Based on the above effort, this work presents the fabrication of highly stable PANI@GO/PEEK membranes taking a bottom‐up integrated approach to achieve the confined polymerization of aniline and the stabilization of multiple interfaces (Figure 1a). First, the commercial GO was dispersed in an aqueous solution to form the uniform brownish‐yellow solution (Figure S5, Supporting Information) and TEM confirmed the GO as the single sheet structure (Figure S6, Supporting Information). The GO/PEEKt membrane was obtained by vacuum‐assisted filtration using the sand core extraction device (Figure S7, Supporting Information). Under the dual action of HCl and (NH_4_)_2_S_2_O_8_, the aniline formed by hydrolysis of the underlying PEEKt diffused from bottom to top (Figure 1a and Figure S8, Supporting Information). The aniline confined polymerizes to form PANI at the inorganic–organic interface, as well as in the GO interlayer. The penetrating insitu growth of PANI well promotes the effective fusion of PEEK with GO and GO interlayer stability, resulting in the formation of the PANI@GO/PEEK membrane. The integrated preparation of the PANI@GO/PEEK membrane realizes high separation efficiency while retaining the flexible characteristics of the polymer membrane (Figure 1b,d).

**Figure 2:**
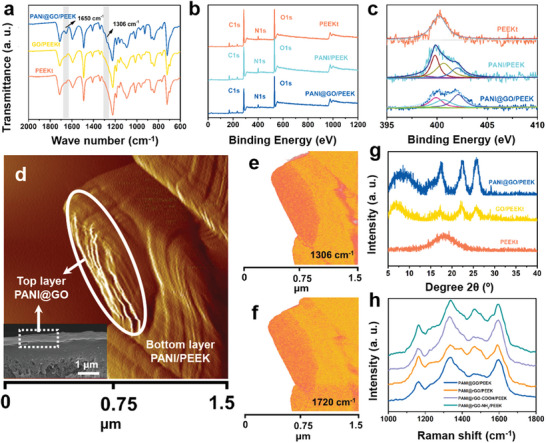
a) ATR‐FTIR spectrum of different membranes. Deconvoluted XPS spectra: b) The XPS survey spectra and c) N 1s of PEEKt, PANI/PEEK, and PANI@GO/PEEK membranes. d) AFM‐phase image (the inset SEM image is the cross‐section of PANI@GO/PEEK membrane), Nano IR, the absorption peak at e) 1306 cm^−1^ and f) 1720 cm^−1^, Nano IR of the cross‐section of PANI@GO/PEEK membrane, Scale bar was 1.5 µm. g) XRD and h) Raman of different membranes.

**Figure** **2**a illustrates the Fourier transform infrared (FTIR) spectra of PANI@GO/PEEK, GO/PEEKt, and PEEKt membranes. Compared with pure GO, the FTIR of PANI@GO/PEEK membrane appeared at the peak of —COOH at 1720 cm^−1^ for —COOH on the GO surface, which confirmed the deposition of GO onto the surface of the membrane.^[^ ^6^ ^]^ Upon hydrolysis with HCl, the ketimine structure was transformed into a ketonic structure and the PEEKt was transformed into PEEK. After treatment with HCl, the C=O vibrational peak at 1650 cm^−1^ appeared in the PANI@GO/PEEK membrane.^[^ ^14^, ^27^ ^]^ This demonstrated the successful preparation of the PEEK substrate. Following the treatment with (NH_4_)_2_S_2_O_8_, the C‐N vibrational peak for N‐Ph‐N appeared at 1306 cm^−1^ for the PANI@GO/PEEK membrane.^[^ ^28^, ^29^, ^30^, ^31^ ^]^ The vibrational peaks of the treated PANI@GO/PEEK membrane matched those of pure PEEK, PANI/PEEK, and GO, demonstrating that the PEEKt membrane was hydrolyzed and oxidized to produce PANI and PEEK. Obtained from the ATR‐FTIR graph, we have fabricated PANI@GO/PEEK membrane successfully.

To further verify the location of the PANI insitu growth process, we have characterized the surface of the membranes with the help of X‐ray photoelectron spectra (XPS). The XPS characterization of the PEEKt membrane, PANI/PEEK membrane, and PANI@GO/PEEK membrane are presented in Figure 2b,c. The full spectrum of XPS shows the presence of three elements of C, N, and O on the three membranes' surfaces. With the XPS curves of N elements, it is evident that the presence of N elements on the GO surface again indicates the successful preparation of PANI. Meanwhile, a detailed analysis of N elements on the membrane surface was performed (Figure 2c). Only the single sub‐peak of N existed on the PEEKt surface and four sub‐peaks of N existed on the PANI/PEEK and PANI@GO/PEEK membrane surfaces. N1s spectra were smoothly fitted to the four sub‐peaks. The 398.1 eV peak is associated with the undoped imine unit. The 399.1 eV peak has the same energy as the previously reported N1s of the undoped imine unit. The 400.9 eV peak is relevant to cationic nitrogen atoms (polaritons and dipoles). The 402.6 eV peak is attributed to protonated amine units.^[^ ^32^, ^33^, ^34^ ^]^ These protonated amine units have higher binding energies. This is due to the conjugation of the sp^3^ bond site resulting in a stronger electronic response. The presence of PANI on the surface of the GO layer was further illustrated by XPS tests, which thereby suggests that the aniline from the underlying layer diffuses to the surface to form PANI. It is again shown that the aniline in the bottom layer diffuses from the bottom up to the surface of the membrane by diffusion and polymerizes to form PANI. This illustrates that the aniline integrates from the bottom to the top to polymerize and form PANI, further stabilizing the GO layer.

To further confirm the state of PANI formation between GO layers during the diffusion of aniline, the PANI@GO/PEEK membrane was sliced at room temperature using the ultra‐thin slicer and characterized by Nano‐IR. As depicted in Figure 2d, the cross‐section of the PANI@GO/PEEK membrane exhibits a distinct internal layer‐by‐layer stacked structure of the GO layer clearly revealing the presence of GO layers.^[^ ^35^, ^36^ ^]^ As shown in the Figure S9, Supporting Information, the IR spectra of the membrane sections showed the Ph—N—Ph peak at 1306 cm^−1^, the C=O peak at 1650 cm^−1^, and the peak of —COOH at 1720 cm^−1^, thus proving that PANI grows within the internal domain of the GO layer.^[^ ^14^, ^31^ ^]^ The Nano‐IR graphs from 1720 and 1306 cm^−1^ further indicate the presence of PANI within the interlayer of GO (Figure 2e,f). While comparing the two graphs, it is evident that PANI is dispersed within the GO layer without agglomeration, indicating that PANI is well dispersed inside the membrane and has no obvious interface with GO. Meanwhile, it is obvious in Figure 2e that PANI is widely dispersed in the top and substrate layers again confirming the ability of PANI to grow at the organic–inorganic interface, which has a significant effect on PANI integration to stabilize multiple interfaces. As the cross‐section of the EDS test shows (Figure S10, Supporting Information), the N element is present in the cross‐section and the surface GO layer has more N elements, thus indicating that more PANI grew in the confined GO layers. Additionally, the EDS results illustrate that the GO layer is not clearly distinguishable from the PEEK layer due to the excellent adhesion effect played by the PANI. The bottom‐up insitu diffusion and polymerization of aniline provide an integrated stabilization way, effectively stabilizing PEEK with GO layer and between the GO layers. The amino group of PANI forms a hydrogen bond with the ketone bond of PEEK, promoting interfacial compatibility. Moreover, the —COOH and —OH of the GO layer formed well hydrogen bonding compatibility with PANI, and there was no obvious interfacial incompatibility during the test. The formation of hydrogen bonding between the two phases effectively hinders the agglomeration of PANI and prevents the agglomeration of PANI caused by too strong hydrogen bonding. The insitu confined polymerization method significantly increased the content of PANI and stabilized the GO and PEEK layers. By characterizing the surface and internal structure of the PANI@GO/PEEK membrane, it was unequivocally confirmed that PANI can be uniformly dispersed throughout the entire membrane to provide an integrated stabilization effect of multiple interfaces.

The 2D material has a stable layer spacing and the layer spacing determines its separation properties. The layer spacing of the membranes was further measured by XRD (Figure 2g). The characteristic absorption peak of the PEEKt polymer at 2*θ* = 17.9°. The characteristic absorption peaks for the non‐woven fabric were at 2*θ* = 17.3°, 22.5°, and 25.7° (Figure S11, Supporting Information).^[^ ^6^, ^28^ ^]^ However, the XRD peaks of the PEEK membrane were partially masked due to the presence of the nonwoven peaks. The characteristic absorption peak of GO/PEEKt appears at 2*θ* = 6.7° compared to PEEKt membrane and non‐woven fabric, which confirmed the existence of GO. For PANI@GO/PEEK membranes, the GO peak becomes larger from 6.7° to 8.5°, which signifies that the average layer distance also known as the d‐spacing of GO decreases from 1.30 to 1.03 nm calculated by Bragg's law.^[^ ^28^ ^]^ This indicates that PANI grows into the interlayer of the GO layer, which further shrinks the layers of the GO due to hydrogen bonding and electrostatic attraction, to provide an accurate separation. To further demonstrate that the growth of PANI did not destroy the structure of the GO layer, Raman's tests were performed (Figure 2h). The D band represents the lattice defects of carbon atoms and the G band represents the stretching motion of carbon atoms sp^2^ in the face. As shown in Figure S12, Supporting Information, the membrane retains the absorption peaks at 1337 and 1596 cm^−1^ compared to the untreated one, thus proving the integrity of the GO layer.^[^ ^7^, ^18^ ^]^ The PANI@GO/PEEK membrane did not destroy the Integrated structure of the GO layer. The D/G of the PANI@GO/PEEK membrane was significantly reduced and the G‐band peak strength was significantly increased when compared to the untreated GO membrane. This suggests that the alignment of GO sheets is more regular, and the repair of GO defects is greatly increased with PANI modification. At the same time, the absorption peaks at 1164 and 1469 cm^−1^ appear, representing the quinonimine structure of PANI, which is another strong proof of the successful preparation of PANI.^[^ ^37^ ^]^ The effective binding of PANI to the GO layer was again confirmed by XRD and Raman, which stabilized the GO layer while maintaining the 2D pore structure of the GO layer.

### 2.2 Microstructure Characterization and Growth of the PANI@GO/PEEK Membrane

**Figure 3:**
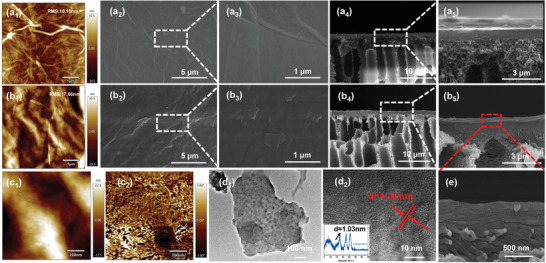
GO/PEEKt membrane of a_1_) the top surface of AFM, a_2_,a_3_) the top surface of SEM images, and a_4_,a_5_) the cross‐section SEM images. PANI@GO/PEEK membrane of b_1_) the top surface of AFM, b_2_,b_3_) the top surface of SEM images, and b_4_,b_5_) the cross‐section of SEM images. The height (c_1_) and the phase (c_2_) of the PANI@GO/PEEK membrane (scale bar was 200 nm). TEM (d_1_), HRTEM (d_2_), and the inset XRD images of PANI@GO/PEEK membrane. e) The cross‐section SEM images of PANI@GO/PEEK membrane (scale bar was 500 nm).

The surface and internal micro‐nano structures of the membranes and the insitu growth process of PANI were investigated by SEM and TEM characterization. The GO surface is the fold structure with stacked sheets, whereas the membrane surface is generally smooth^[^ ^19^, ^20^ ^]^ (**Figure** **3**a_2_,_3_). From the cross‐section of the GO/PEEKt membrane, it can be seen that the bottom part of the membrane is a finger‐like pore structure and the top of the membrane is dense^[^ ^14^ ^]^ (Figure 3a_4_,_5_). It can be evidenced that the GO membrane layer is a stacked structure of layers, and the membrane thickness is about 200 nm. Compared with the GO/PEEKt membrane, the surface of the PANI@GO/PEEK membrane is consistent with the 2D membrane, both are uniform and defect‐free layer‐stacking structures (Figure 3b_2_,_3_). While there are minuscule particles on the surface of the membrane, it is clear that PANI obviously modified the surface of the GO membrane. From the cross‐section of the PANI@GO/PEEK membrane, it can be seen that the bottom part of the membrane is a finger‐like pore structure, and the top part is a lamellar structure of GO by layer stacking with a thickness of about 250 nm (Figure 3b_4_,_5_,e). As the figure shows, the cross‐section sheets are rougher and the thickness increases significantly, which can be explained that PANI grows in the interlayer of the GO layer and increases the thickness of the GO layer. Meanwhile, no obvious organic–inorganic interface was observed, due to the presence of PANI effectively linking the two phases. Finally, the growth of PANI promoted the formation of a stable integrated interfacial structure between GO and PEEK, which again confirmed the growth of PANI between GO and PEEK. Compared with the PANI/PEEK membrane, the surface of the membrane is a granular structure of PANI, which is consistent with the growth of the GO layer (Figure S13, Supporting Information). AFM tests were performed to analyze the roughness of the membrane surface. It was observed from the membrane surface that the surface of the PANI@GO/PEEK membrane was rougher compared to the pure GO membrane due to the addition of PANI, with the roughness shifting from 10.16 to 17.66 nm (Figure 3a_1_,b_1_). The surface phase separation from the small size showed the presence of small aggregates of PANI and the presence of small particles on the membrane surface (Figure 3c_1_,_2_). Further exploring the growth process of PANI inside the membrane, small particles appeared inside the pure GO sheets according to TEM (Figure 3d_1_). It can be proved that PANI grows not only in the GO layers but also between PEEK and GO layers. From HRTEM, the layer structure of GO layers with the layer spacing of 1.05 nm is consistent with XRD (Figure 3d_2_). This further indicates that PANI is grown in the interlayer of GO layers, which has a significant effect on stabilizing the layer spacing. The characterization of the microscopic morphology of PANI@GO/PEEK membranes confirmed that PANI has grown in a homogeneous manner throughout the system. Meanwhile, the homogeneous alteration of PANI achieved the modulation of the micro‐nano structure morphology of the membrane surface and the well‐repaired multiple interfaces. The integrated growth of PANI promoted the integrity of membrane structure and the remediation of defects inside the membrane.

### 2.3 Growth Mechanism of the PANI@GO/PEEK Membrane

To gain further insights into the integrated stabilization of multiple interfaces by the PANI and the formation process of PANI@GO/PEEK membrane, the comprehensive investigation was conducted on the confined polymerization of PANI, and the underlying mechanism of the whole experimental process was investigated. Aniline is obtained from the bottom PEEKt precursor by hydrolysis and diffuses from the lower substrate layer to the top GO layer interlayer. Remarkably, aniline self‐polymerizes at the organic–inorganic interface, as well as the GO layer. Regarding the bottom‐up diffusion of aniline, it first diffuses at the organic–inorganic interface, where PANI relies on van der Waals force interactions to link PEEK and GO to form the stable interface. Subsequently, the aniline continues to diffuse toward the upper layer, and under the influence of multiple effects, the PANI stabilizes the GO layer while effectively repairing the defects of GO layer stacking.

**Figure 4:**
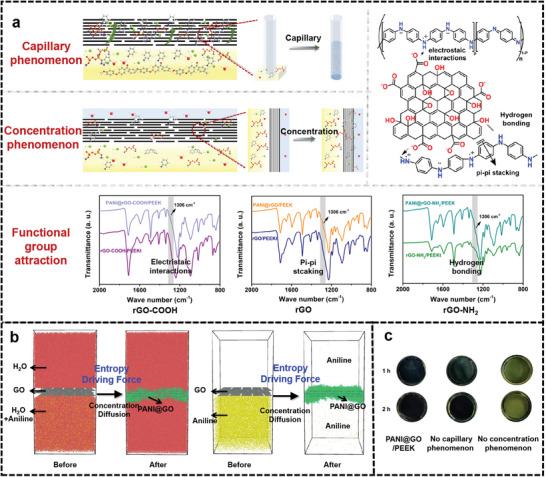
a) Synthesis mechanism and growth factors of PANI@GO/PEEK membrane. b) MD simulation diagrams of aniline before and after the concentration effect diffusion and bottom‐up polymerization in the GO layer. c) Physical images of PANI@GO/PEEK membrane with different reaction processes.

The diffusion of aniline into the GO layer is influenced by several factors, including capillary phenomena, concentration, and functional group attraction, as demonstrated in **Figure** **4**a and Figure S14, Supporting Information. The capillary phenomenon plays a significant role in this process. Layers of stacked GO have obvious capillary pore channels between the layer structure of the sheets. During the membrane formation process, there is a period of resting volatilization process for a certain time during the formation of the membrane (Figures S8 and S14, Supporting Information). The solution at the top of the membrane volatilizes the solution underneath due to the existence of capillary phenomenon, allowing the aniline at the bottom layer after hydrolysis diffuses to the GO layer from the bottom to the top together with water. The concentration difference effect is another influential factor. In the final stage of membrane preparation, ultrapure water is present at the top of the membrane. The ultrapure water at the top (low concentration) and the solution at the bottom (high concentration) form the significant concentration difference effect with the isolation of the GO layer. The diffusion from the side of high concentration to the side of low concentration is accompanied by the diffusion of aniline from the bottom layer to the top layer. To illustrate the importance of these factors, the physical diagram of the reaction process was examined under conditions without capillary action (no natural volatilization process) and without concentration effect (high concentration solution at the top). As depicted in Figure 4c, PANI@GO/PEEK membrane could not achieve growth well and exhibited noticeable defects in these scenarios. Based on the capillary phenomenon and the concentration difference effect, the aniline from the bottom layer diffuses to the top GO layer and polymerizes to form PANI in the confined interlayer under the action of HCl and (NH_4_)_2_S_2_O_8_. In addition, the concentration difference effect exhibits a more significant influence on the aniline diffusion from the experiment. To further explore the bottom‐up diffusion of aniline within the GO layer under the influence of the concentration difference effect, molecular dynamics (MD) simulations were conducted^[^ ^4^, ^16^, ^22^ ^]^ (Figure 4b and Figure S15, Supporting Information). Before and after the simulated diffusion of aniline, it is obvious that aniline diffuses from the bottom to the top within the GO layer and forms a stable PANI structure within the GO layer, as well as on the surface. The entropy‐driven effect of the concentration difference diffusion achieved the bottom‐up diffusion of aniline. Concurrently, the aniline was polymerized while diffusing by the presence of (NH_4_)_2_S_2_O_8_ and HCl in the membrane. Finally, the effective bonding of PANI to the GO layer is formed by the attraction of the GO functional group, which efficiently repairs defects in the GO layer. It was confirmed again that the presence of the entropy driving force promoted the concentration diffusion effect of aniline, enabling the process of aniline diffusing from bottom to top in the GO confined space.

After attracting to the interlayer of the GO layer, there are obvious van der Waals forces due to the existence of aniline and PANI with the oxygen‐containing functional groups of GO sheets (Figure 4a and Figure S14, Supporting Information). The *π*–*π* interaction of PANI and GO, as well as the electrostatic attraction of positively charged PANI with —COOH and the hydrogen bonding interaction of the amino group of aniline with —OH and —COOH groups, further promote the diffusion of aniline into the interlayer of GO, forming the effect of PANI insertion into the interlayer of GO to stabilize the GO layer. To verify the existence of van der Waals forces, reduced graphene oxide (rGO) with different groups was employed. The above van der Waals force interactions were verified using rGO, rGO‐COOH, and rGO‐NH_2_. It is obvious from the FTIR, Raman, XPS, and SEM variations that PANI grows between the layers of GO due to different van der Waals forces attraction. The FTIR pattern displays the clear ph‐N‐ph characteristic absorption peak at 1306 cm^−1^ for PANI. (Figure 4a) The N‐spectrum curves of XPS are consistent with PANI@GO/PEEK membranes which can be divided into four characteristic absorption peaks (Figure S16, Supporting Information). Raman spectrum also shows the characteristic absorption peak of the quinonimine structure of PANI at 1164 and 1469 cm^−1^ (Figure 2h and Figure S12, Supporting Information). These findings support the in situ confined polymerization of PANI can be carried out inside and on the surface of rGO with different functional groups. The surface SEM shows that there is a layer stacking on the surface of the membrane along with the modification of PANI particles on the surface (Figure S17, Supporting Information). It can be concluded that the capillary, the concentration difference effect, and the attraction of van der Waals force functional groups mutually promote the autonomous diffusion of aniline. The bottom‐up diffusion of aniline achieves homogeneous binding of PANI, while insitu confined polymerization of PANI serves to anchor multiple interfaces in an integrated manner.

The integrated growth of PANI was achieved by a combination of factors including HCl, (NH_4_)_2_S_2_O_8_, and GO layer thickness. Aniline is obtained by hydrolysis of PEEKt. Therefore, when the HCl concentration is excessively high, the underlying aniline polymerizes in large quantities at the interface between the PEEK and GO layers. The entropic driving force cannot be counteracted by the interfacial resistance of the GO layer, causing the aniline to be unable to diffuse upward effectively eventually leading to the rupture of the top GO layer (Figure S18, Supporting Information). Similarly, a high concentration of (NH_4_)_2_S_2_O_8_ leads to significant polymerization of aniline within the GO layer. Therefore, it is crucial to regulate the influence of different conditions during the growth process for the formation of highly stable structures in PANI@GO/PEEK membranes.

### 2.4 The Influence of Different Conditions and Organic Solvents Nanofiltration Performance for PANI@GO/PEEK Membrane

Solvent flux and rejection are important factors in separation membranes, but there is a trade‐off effect between them during the actual separation process. Therefore, modulating different variables to change the structure of the nano‐separation layer is an important factor in regulating the membrane. For further investigation of the effects of different conditions on the formation of PANI@GO/PEEK membranes and the changes in microscopic morphology during their formation, water flux, the rejection of rose bengal sodium (RB) in water, XRD, and SEM tests were performed. The flux of the membrane was tested by a self‐made cross‐flow filtration device (Figure S19, Supporting Information), and the rejection of the aqueous solution of RB of the membrane was also tested by a self‐made dead‐end filtration device^[^ ^7^, ^12^, ^14^ ^]^ (Figure S20, Supporting Information). Herein, the effect of c((NH_4_)_2_S_2_O_8_), c(HCl), and m(GO) was explored for the membrane formation on a case‐by‐case basis (Tables S2–S4, Supporting Information).

**Figure 5:**
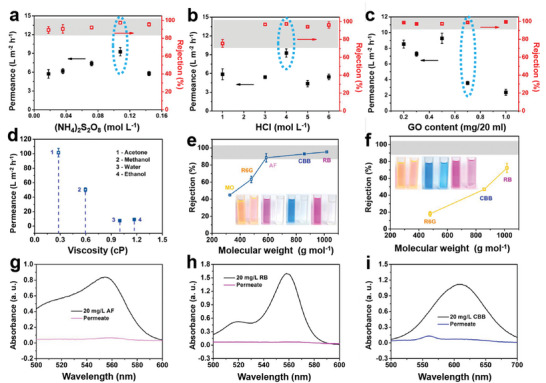
Water flux and RB rejection in the pure water of PANI@GO/PEEK membranes with different treatments a) c[(NH_4_)_2_S_2_O_8_] from 0.036 to 0.144 mol L^−1^, b) c(HCl) from 1 to 6 mol L^−1^, and c) m(GO) from 0.3 to 1 mg at 6 bar. d) The plot of solvent permeance versus the viscosity of the solvent of PANI@GO/PEEK membranes. Dyes rejection of e) PANI@GO/PEEK and f) GO/PEEKt membranes in ethanol. g–i) PANI@GO/PEEK membranes UV–vis spectra of feed dye and permeate.

With the increase of c((NH_4_)_2_S_2_O_8_), the flux of the membrane increases and then decreases, while the rejection increases and then remains constant (**Figure** **5**a). The XRD shows that the 2*θ* increases and then decreases, and the layer spacing (*d*) decreases and then increases (Figure S21, Supporting Information). As the c((NH_4_)_2_S_2_O_8_) directly affects the growth content of PANI, with the increase of PANI content, the layer spacing of GO decreases, and the flux increases due to the van der Waals force. As the c((NH_4_)_2_S_2_O_8_) increases, the PANI content increases and the PANI accumulates in the layers of GO, causing the layer spacing of GO to become larger. It is obvious from Figure S22, Supporting Information, that the particles on the surface gradually increase and the interlayer gradually becomes rough, which again proves that (NH_4_)_2_S_2_O_8_ promotes the growth of PANI in the interlayer.

Since the prepared PANI was polymerized insitu due to the diffusion of the bottom aniline into the interlayer, the aniline produced was limited by the c(HCl) of the bottom PEEKt. As the concentration of c(HCl) increases, the flux of the membrane increases and then decreases, whereas the rejection increases and then remains constant. At low c(HCl), the generation of aniline from the bottom layer is slower, and consequently, less aniline diffuses into the GO layer. Less PANI is produced which cannot plug the defects in the GO layer and stabilize the GO layer resulting in the water flux and rejection being low (Figure 5b). With the increase of c(HCl), the aniline can be more dispersed in the GO layer, which has a better stabilization effect. But the more aniline in the bottom layer generated with excessive c(HCl), PANI is formed faster and polymerized more in the confined GO sheets. GO layer spacing did not change significantly (Figure S23, Supporting Information). As shown in the Figure S24, Supporting Information, the interlayer particles are obviously increased and there exists the problem of PANI agglomeration. At the same time, due to the faster generation of aniline in the bottom layer, more PANI is formed at the bottom layer, and there will be a problem that the GO layer sheets are destroyed. So consistently, the c(HCl) further regulated the diffusion and generation rate of aniline in the bottom layer, which played an important role in stabilizing the GO layer and constructing the PANI@GO/PEEK membrane.

**Figure 6:**
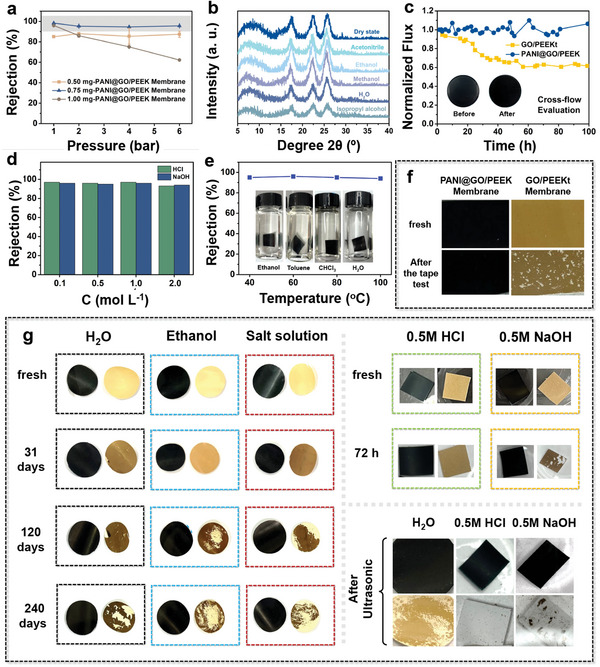
a) The rejection of RB of PANI@GO/PEEK membranes with different treatment m(GO) under different operating pressures in ethanol. b) XRD patterns of the PANI@GO/PEEK membrane, showing the interlayer spacing between GO nanosheets when dried, as well as in water and a multitude of organic solvents. c) The unimpaired integrity after the cross‐flow evaluation is also substantiated by the long‐term cross‐flow stability of the PANI@GO/PEEK membrane, as demonstrated by the stable water permeance. d) The rejection of RB in m NaOH and the rejection of AF in m HCl of PANI@GO/PEEK membranes. e) The rejection of RB of PANI@GO/PEEK membranes in ethanol at room temperature, which was soaked in toluene at different temperatures for 24 h (the inset physical image is PANI@GO/PEEK membrane that soaked in solvents at 60 °C for 72 h). f) Photographs of the PANI@GO/PEEK and GO/PEEKt membranes before and after the tape test. g) Physical images of GO/PEEKt membrane and PANI@GO/PEEK membrane soaked in water, ethanol, salt solution, 0.5 m HCl, and NaOH. Physical images of GO membrane and PANI@GO/PEEK membrane treated with ultrasonic treatment (80 kHz) of membranes. (Black membrane is PANI@GO/PEEK membrane, yellow membrane is GO/PEEKt membrane)

The thickness of the GO layer has a significant effect on the water flux and rejection of the membrane (Figure 5c). m(GO) is an important factor in determining the thickness of the GO layer deposited. As the deposition thickness increases, the flux of the membrane increases and then decreases, and the rejection remains the same basically. With m(GO) deposition thicker, the thickness of the top layer of the membrane increases from 100 to 500 nm as seen from SEM (Figure S26, Supporting Information). The GO layer is stacked more closely for the denser separation layer, therefore the flux decreases while the rejection increases. When the thickness is thinner, PANI diffuses more in the GO layer causing the layer spacing to increase. When the GO layer is thicker, the aniline diffusion resistance is higher and PANI cannot effectively enter the GO layer causing the layer spacing to be almost the same as pure GO (Figure S25, Supporting Information). Too thick GO layer will cause PANI to be unable to enter the interlayer effectively, which will not provide a better stabilization effect. In addition, PANI@GO/PEEK membranes were tested with the rejection of RB in ethanol under different pressures to examine the stability. From **Figure** **6**a, the rejection of RB at 0.5 mg is at 85% due to less lamellar structure. When the deposition quantity increases, the membrane interception decreases with increasing pressure because of the greater rigidity of GO and the increased resistance to aniline diffusion, and the 1 mg‐PANI@GO/PEEK membrane has a situation of membrane rupture with increasing pressure. Therefore, 0.75 mg‐PANI@GO/PEEK membrane was selected to test the performance of OSN performance. 0.75 mg‐PANI@GO/PEEK membrane was able to maintain excellent stability and 95% of RB rejection in ethanol at different pressures.

It can be clearly seen that the c((NH_4_)_2_S_2_O_8_), c(HCl), and m(GO) are synergistically influencing the formation of the membrane, which has a significant effect on the microscopic layer spacing, micro‐nano structure, and separation performance. The effects on membrane stability, as well as properties, were further clarified by modulating the effects of different factors in the preparation of PANI@GO/PEEK membranes. The variation pattern of different factors on membrane formation was also investigated, which has a significant impact on obtaining PANI@GO/PEEK membranes with excellent properties.

The OSN separation performance of the PANI@GO/PEEK membrane was evaluated by self‐made dead‐end filtration due to the ease and simplicity of the method.^[^ ^6^ ^]^ First, we evaluated the permeability of water and various common organic solvents. Usually, the permeability of the membrane depends mainly on the viscosity of the solvent.^[^ ^4^, ^23^ ^]^ This dependence can be illustrated by the Hagen–Poiseuille equation, which shows an approximately inverse decrease in permeability with increasing viscosity. The test results for PANI@GO/PEEK membranes are consistent with those reported in the literature^[^ ^2^, ^4^ ^]^ (Figure 5d). The flux of methanol was able to be maintained at 50.2 L m^−2^ h^−1^. This indicates that the PANI@GO/PEEK membrane does not destroy the original 2D structure of GO. Flux and rejection are always important factors in the evaluation of membrane performance. The membrane selectivity was evaluated with the rejection of different dyes. Methyl orange (MO; 327.33 g mol^−1^), Rhodamine 6G (R6G; 479.01 g mol^−1^), acid magenta (AF; 585.53 g mol^−1^), Coomassie brilliant blue (CBB; 858.05 g mol^−1^), and RB (1017 g mol^−1^) were dissolved in ethanol for selectivity measurements (Table S5, Supporting Information). The PANI@GO/PEEK membrane was able to maintain a higher retention rate compared to the pure GO membrane (GO/PEEKt membrane) (Figure 5e,f). At the same time, efficient rejection of small molecule dyes was achieved. For AF (585 g mol^−1^), it was able to maintain a level of more than 90%. The UV test confirmed this conclusion again (Figure 5g–i and Figures S27 and S28, Supporting Information). This confirmed the ability of PANI to efficiently stabilize the GO layer while filling the defects of the GO layer. This could further indicate that the PANI@GO/PEEK membrane achieves efficient repair of GO layer defects and achieves a high level of interception of small molecules while maintaining the high flux. Compared with other organic solvent separation membranes, the PANI@GO/PEEK membrane obtained in this work can maintain higher methanol flux and smaller molecular weight rejection, which is significant for the fabrication of OSN membranes (Figure S29, Supporting Information).

### 2.5 Stability and Durability of the Membrane

For 2D membrane materials with the layer structure of GO, the stability and long‐term durability of the membrane is an important condition that limits the membrane separation performance. First of all, the rejection of RB in ethanol and its variation with pressure for PANI@GO/PEEK membranes were tested with different deposited thicknesses of GO. From Figure 6a, the 0.75 mg‐PANI@GO/PEEK membrane was able to maintain excellent stability and 95% of RB rejection in ethanol at different pressures, which confirmed the pressure stability of the membrane during use. To further verify the swelling behavior of GO in different solutions, the wet membranes soaked in different solutions were characterized by XRD. It can be seen from Figure 6b that the membranes have excellent stability and the 2*θ* does not change significantly compared to the dry membranes, with a variation range of about 1°. This is due to the fact that PANI grows between GO sheets and is effective in limiting the swelling of the GO layer when the solvent enters between the layers of the membrane. Meanwhile, we tested the long‐term stability of PANI@GO/PEEK membranes by using a self‐made cross‐flow test machine at a pressure of 6 bar. PANI@GO/PEEK membranes maintained long‐term durability of 100 h. This is of great significance in demonstrating the large‐scale long‐term use of the membranes. The membrane surface did not change significantly before and after the test. However, the pure GO membrane has severe swelling behavior and the flux decreases significantly (Figure 6c).

The membrane separation process usually occurs in extreme environments. The operating state of membranes under extreme conditions is also an important factor limiting the stability of membranes. 2D membranes often break and fall off under high temperature, acid, or base environments. Therefore, it is more than important to explore the stability of the membrane under extreme conditions. By exploring the separation properties of RB and AF under acid or base conditions, it has been successfully demonstrated that PANI@GO/PEEK can maintain a stable state and maintain a high rejection of 90% (Figure 6d). At the same time, compared with the GO membrane, we found that the GO membrane had an obvious shedding phenomenon after 72 h immersion (Figure 6g). Under acid–base conditions, the GO membrane was completely detached from the basement membrane and could not be used whereas the PANI@GO/PEEK membrane remained unchanged. Second, the high‐temperature stability of the membrane was further evaluated. Soaked the membrane in toluene at different temperatures for 24 h and then tested the retention of RB in ethanol at room temperature. PANI@GO/PEEK membrane still maintained a high rejection of more than 95% (Figure 6e). At the same time, the membrane still remained unchanged when soaked in different solutions at 60 °C for 72 h. It is confirmed again that PANI@GO/PEEK membranes had excellent stability, which maintained a high level for the long‐term use and stability of the membrane. The PANI@GO/PEEK membrane retains its original morphology on the membrane surface after sonication due to the stability of PANI and the good compatibility of PANI and PEEK substrate layers during in situ confined growth. Maintaining the stability of adhesion between the GO layer and the substrate layer is also an important factor in evaluating membrane stability. The two membranes were compared by using the tape adhesion method (Figure 6f). Due to the lack of stability at the organic–inorganic interface, the GO/PEEKt membrane suffered from large areas of peeling. However, PANI@GO/PEEK membranes remained in a better condition with little peeling behavior. This confirms that the PANI@GO/PEEK membrane with integrated PANI growth plays a strong bonding effect in stabilizing the organic–inorganic boundary layer. For demonstrating membrane durability, PANI@GO/PEEK membranes and pure GO/PEEKt membranes were immersed in ultra‐pure water, salt water, ethanol, 0.5 m HCl, and 0.5 m NaOH. It can be seen that the pure GO membranes showed subtle changes on the membrane surface after 7 days of immersion and significant damage on the membrane surface after 240 days of immersion (Figure 6g and Figure S30, Supporting Information). In contrast to PANI@GO/PEEK membranes, no significant damage was found on the membrane surface after 240 days of immersion in different solutions, and remain the whole membrane structure. Meanwhile, the stability of PANI@GO/PEEK membranes was further analyzed using ultrasonic treatment. The PANI@GO/PEEK membrane, and GO/PEEKt membrane were ultrasonicated at 80 Hz, and thus the stability of the membranes was assessed. As can be seen in Figure 6g, the pure GO membrane surface showed significant damage, and the GO layer could be seen to peel off from the membrane surface. The above experiments also confirmed the solvent and corrosion resistance of PEEK and its stability as a base material during long‐term use. This integrated construction of special separation membranes enables the complete connection from the substrate, interface to the precise separation layer, which effective construction of solvent‐resistant PEEK substrates and GO interfaces. This work strongly illustrates the super stability and durability of PANI@GO/PEEK membranes, which achieve effective stabilization of GO layers, the corrosion resistance of PEEK, and efficient bonding at the organic–inorganic interface. The integrated growth of PANI‐stabilized multiple interfaces proposed in this work has a significant effect on the stable and durable performance of 2D materials.

### 2.6 Universality of 2D Membrane

GO is widely used in the field of separation membranes because of its regular pore structure. However, GO suffers from problems, such as swelling and easy breakage, so stabilizing 2D membrane materials has become a hot research topic. In previous researches, the use of metal cross‐linking, polymer cross‐linking, particle filling, and filling of small‐diameter lamellar materials have provided well‐stable effects for stabilizing GO layers.^[^ ^4^, ^18^, ^19^, ^20^, ^21^, ^22^, ^23^, ^24^ ^]^ However, the high price, as well as the high rigidity of 2D materials, currently make it difficult to be used alone in the separation field. Commercially available polymer membranes are usually used as substrate membranes for separations. Therefore, we believe that the well bonding of the substrate membrane to the GO layer is also an important factor in evaluating the membrane stability. In the majority of previous reports, vacuum‐assisted filtration is usually used to attach the substrate membrane to the GO layer (Table S6, Supporting Information).^[4, 15–20, 22–24, 38–43]^ We consider that this may have the possibility of detachment of the separated layer. Therefore, the integrated preparation of the PANI@GO/PEEK membrane proposed in this work effectively solves the problem of incompatible organic–inorganic interfaces. The bottom‐up growth of PANI stabilizes multiple interfaces and presents an innovative new idea for GO separation. At the same time, compared with the traditional method of post‐modification of the substrate membrane, the method proposed in this work is simple and efficient, while achieving the integrated growth has a significant effect on the stability and durability of the interface.

The integrated stabilization method proposed in this work achieves the improvement of the stability of the separation membrane for the 2D material represented by GO. This work innovates a new idea of separation membrane preparation. The method has excellent universality and application prospects for stabilizing 2D materials. For 2D materials, such as GO, MXene, rGO‐COOH, rGO, and MoS_2_, the corresponding effect can be achieved by this method (Figure S31, Supporting Information). The simple and efficient integrated growth mode is used to stabilize the multiple interfaces while achieving an efficient application of OSN. This work has a profound impact on the application of 2D materials in the field of separation and their application in industrial production. Meanwhile, PANI has good electrical conductivity, and the PANI films prepared by this method have potential applications in supercapacitors and flexible electronic devices.

## 3 Conclusion

Overall, highly stable and durable corrosion‐resistant PANI@GO/PEEK special separation membranes were successfully fabricated through integrated PANI polymerization to stabilize multiple interfaces. The method employed in this work effectively stabilized the organic–inorganic interface and the GO interlayer was achieved by modulating the bottom‐up diffusion of aniline by insitu confined polymerization. The method used in this work achieves that the GO layer is uniformly covered and defects are effectively repaired while enhancing the adhesion of the GO and substrate in a simple and efficient manner. The homogeneous modification of PANI achieves the modulation of the surface micro‐nano structure of the PANI@GO/PEEK membrane, enabling the conversion of solvent‐resistant PEEK substrates. Based on the integrated growth of PANI, the membrane generation mechanism is proposed and verified in this work. Owing to the capillary phenomenon, concentration phenomenon, and van der Waals force interaction, insitu diffusion of aniline and confined polymerization within the layer of GO are realized. Highly stable PANI@GO/PEEK membranes were also obtained by modulating different variables, and the various patterns of different variables were explored. The membrane maintained a high methanol permeability of 50.2 L m^−2^ h^−1^, as well as high selectivity for over 90% rejection of AF (585 g mol^−1^). Furthermore, PANI@GO/PEEK membrane was able to maintain the original performance after 240 days of immersion and 100 h of long‐time operation, showing super high durability. The membrane exhibited exceptional stability and high rejection of more than 90% under harsh conditions, including exposure to 2 m HCl, NaOH, and high temperature, which maintains structural integrity under ultrasound and friction. This work proposes an integrated method to stabilize multiple interfaces for the separation and durability of special separation membranes with significant application effects, which provides a powerful approach for the practical application of 2D membranes.

## 4 Experimental Section

Detailed experimental information is reflected in the Supporting Information

## Conflict of Interest

The authors declare no conflict of interest.

## Supporting information

Supporting Information

## Data Availability

The data that support the findings of this study are available from the corresponding author upon reasonable request.
