# Supplementary material for: InSitu Integrated Fabrication for Multi‐Interface Stabilized and Highly Durable Polyaniline@Graphene Oxide/Polyether Ether Ketone Special Separation Membranes
Source: Adv Sci (Weinh). 2023 Jun 28;10(25):2302654. doi: 10.1002/advs.202302654 (PMC10477839; doi:10.1002/advs.202302654)
Supplement: Supplementary file 1 — Supporting Information [file ADVS-10-2302654-s001.pdf]

ADVANCED SCIENCE
Open Access

Supporting Information

for *Adv. Sci.*, DOI 10.1002/advs.202302654

InSitu Integrated Fabrication for Multi-Interface Stabilized and Highly Durable Polyaniline@Graphene Oxide/Polyether Ether Ketone Special Separation Membranes

Ziyu Lin, Jundong Zhong, Runyin Sun, Yingzhen Wei, Zhonghui Sun, Wenying Li, Liyuan Chen, Yirong Sun, Haibo Zhang, Jinhui Pang and Zhenhua Jiang*

Supporting Information

In-situ Integrated Fabrication for Multi-interface Stabilized and Highly Durable Polyaniline@Graphene Oxide/Polyether ether ketone Special Separation Membranes

Ziyu Lin[1], Jundong Zhong[1], Runyin Sun[1], Yingzhen Wei[1], Zhonghui Sun[1], Wenying Li[1], Liyuan Chen[1], Yirong Sun[1], Haibo Zhang[1], Jinhui Pang[1] and Zhenhua Jiang[1]*

[1*]Key Laboratory of High Performance Plastics (Jilin University), Ministry of Education, National & Local Joint Engineering Laboratory for Synthetic Technology of High Performance Polymer, College of Chemistry, Jilin University. Jilin University, Changchun 130012, People's Republic of China.

* Corresponding Authors: pangjinhui@jlu.edu.cn.

1 Chemicals and Materials

Ammonium persulfate ($(NH_4)_2S_2O_8$), hydroquinone, 4,4'-difluorobenzophenone, molecular sieves (4 Å), sulfolane and aniline were purchased from Shanghai Aladdin Co., Ltd., China. Hydrochloric acid (HCl), N-methylpyrrolidone (NMP), potassium carbonate (K_2CO_3), methanol, ethanol and Rose Bengal sodium (RB) were purchased from China Xilong Science Co., Ltd. Graphene oxide (GO), graphene (rGO), aminated graphene (rGO-NH_2), carboxylated graphene (rGO-COOH), Mxene, MoS_2 were purchased from Nanjing Pioneer Nanotechnology Co., Ltd., China. All reagents and solutions were not purified before use.

2 Characterization methods

2.1 Nuclear Magnetic Resonance Spectrometer (NMR)

The monomers and polymers were dissolved in deuterated DMSO and passed through a nuclear magnetic resonance spectrometer (model Avance NEO, purchased from Swiss BRUKER Instrument Company, and the test condition was 400 Hz)

2.2 Fourier transform infrared spectrometer (FTIR)

The functional groups of all materials are tested by a Fourier transform infrared spectrometer, the instrument model is iS10, purchased from Thermo Fisher Scientific, which the test mold is in total reflection mode.

2.3 Nano infrared system (Nano-IR)

The nano-infrared spectrum and the cross-section of AFM phase diagram of the PANI@GO/PEEK membrane section were tested by nano-infrared system, the instrument model is NanoIR3, purchased from Bruker. The section of the PANI@GO/PEEK membrane was sliced by cryostat under normal temperature mode, and the slice thickness was 500 nm.

2.4 Wide-angle X-ray diffractometer (WXRD)

Wide-angle X-ray diffractometer (WXRD) were analyzed by Empyrean purchased from PANalytical B.V. The 2θ scan data were collected at 6 ^{o}/min intervals over ranges of 10°–50°.

XRD calculation layer spacing is calculated by Formula (S1):

$$2d\sin\theta = n\lambda$$

Where the d: distance between crystal planes; θ: read out on the picture; x-ray wavelength n=1; λ=0.15406 nm

2.5 X-ray photoelectron spectra (XPS)

The surface structure of membranes was analyzed by XPS. X-ray photoelectron (XPS) spectra were collected using a Thermo ESCALAB 250 equipped with an Al Ka X-ray source (1486.6 eV).

2.6 Transmission electron microscope (TEM)

The internal structure of different GO nanosheets was tested by TEM. The instrument model was Tecnai G2 S-Twin F20, purchased from FEI ELECTRON OPTICS in the Netherlands.

2.7 High resolution transmission electron microscope (HRTEM)

The lattice structure of the PANI@GO/PEEK membrane was tested by HR-TEM, the instrument model was FEI Tecnai G2 F20, purchased from FEI Company in the United States.

2.8 Scanning electron microscope (SEM)

The surface morphology of the sample was observed by the scanning electron microscope (SEM/FEI company, USA, Model Nova nano 450). Before testing, the sample was sprayed with Platinum for 60 seconds. The cross section was that the membranes were quenched in liquid nitrogen.

2.9 Frozen Ultrathin Microtome

The membrane was sliced to prepare a test sample. The membrane was wrapped in epoxy resin, and sliced with a Leica cryo-microtome, the instrument model was EM FC7, which was purchased from Leica Company.

2.10 Energy Dispersive Spectrometer (EDS)

The elements of of the sample was observed by the scanning electron microscope (SEM/FEI company, USA, Model Nova nano 450). Before testing, the sample was sprayed with Platinum for 60 seconds. The cross section was that the membranes were quenched in liquid nitrogen.

2.11 Raman spectroscopy

High-Resolution Laser Raman Spectrometer (Horiba, LabRAM HR Evolution) was used to detect the D band and G band of carbon atoms. A 632nm laser was used.

2.12 Atomic Force Microscope (AFM)

Cut the membrane into 1cm*1cm square samples, and stick the membrane on the sample stage. Asylum Research Cypher ES was used to take the AFM images.

2.13 Tape testing

We conducted a tape test, based on the ASTM D3359-Standard Test Methods for Measuring Adhesion by Tape Test. Adhesion was tested using Scotch tape. The adhesion of the GO membrane was assessed by applying and removing tape on the GO membrane: (i) fix the membrane on a circular glass plate to provide a sufficiently flat test area; (ii) with a purge gas to clean the GO surface slightly; (iii) Put a piece of tape on the GO layer, wipe the tape vigorously with fingers to make sure the tape is in good contact with the membrane; (iv) After 60 seconds, grab the free end to quickly remove the tape; (v) Visually inspect the adhered area for removal of the coating from the substrate. To ensure the accuracy of this test, multiple repetitions of the same sample were performed.

2.14 Long-term immersion testing

The GO/PEEKt and PANI@GO/PEEK membrane was immersed in pure water, ethanol, 1 mg/L NaCl solution, 0.5M HCl and 0.5M NaOH, and its stability was evaluated by long-term immersion. Its long-term stability was confirmed by photographing.

2.15 Ultrasound testing

The membrane was sonicated at 80 Hz to test its stability. The stability was evaluated by observing the state of the membrane surface.

2.16 Long term stability testing

The membranes were placed in a self-made cross-flow test cell for long-term stability testing with a pressure of 6 bar. The long-term stability was evaluated by observing the membrane surface state and water flux changes.

2.17 Water flux testing of membranes

The water flux of the membrane was measured by a self-made cross-flow filtration device. The measuring equipment includes a solution storage tank, a cross-flow device with the diameter of 3 cm, and water pump. Before testing, the membrane was placed in a cross-flow device, and the pressure was adjusted to 0.8 MPa for 60 min. During the test, the pressure was adjusted to 0.6 MPa and counted every 15 min for 120 min. Each membrane was measured 4 times in parallel. The pure water flux of the membranes (J_w, L m^{-1} h^{-1}) was defined as follows the Formula (S2):

$$Jw = \frac{V}{A * \Delta t}$$

where V (L) was the permeate volume; A (m^2) was the effective filtration area; t (h) was the collection time.

2.18 The rejection test of membranes

Solvent flux and rejection tests for membranes were measured with a self-made dead-end filtration device. The measuring equipment includes a dead-end filter cell with the diameter of 2 cm, gas-controlled pressure driver, and liquid storage tank. Before the test, adjust the pressure

to 0.6 MPa for 30 min, and adjust the pressure to 0.5 MPa during the test. Each membrane was measured 4 times in parallel.

Solvent flux was calculated using Formula (S2).

The self-made experiment of the membrane was measured by configuring the 20 mg/L of different dyes ethanol solution. The rejection of the membrane is measured by the following Formula (S3):

$$R = \left(\frac{Cp}{Cf}\right) * 100\%$$

where Cp and Cf were the concentrations of dyes in the permeate and in the feed respectively, as analyzed via UV–vis spectrophotometry (UV-2501, Shimadzu, Japan).

2.19 Theoretical simulation details.

2.19.1 The DPD method

Since the atoms are vast in amount in this system, it is inevitable to lose some resolution for obtaining sufficient basic data. As for our system, the diffusion and polymerization of monomer are much more crucial, while details about electrons and particular atoms is not noteworthy in this system. Herein, the coarse-grained (CG) model is utilized in our Molecular Dynamics (MD) simulations [S1-S4].

For simplicity, we build the CG model containing five types of beads, that is, A, G, P, S (**Figure S17**), where A represents the monomer (normal aniline), G represents the unit of Graphene Oxide (a lump of six carbon atoms), P represents the repeat unit of polyaniline, P* represents the initiator (aniline molecule with free radical), S represents solvent (a lump of four water molecules), respectively. In order to illustrate the diffusion and polymerization of monomer along with time in this study, the dissipative particle dynamics (DPD) force field is adopted for describing the interaction between different beads.

DPD simulation is about the evolution of the particle's momentum and position along with time, and governed by Newton's equations of motion. Interparticle interactions are characterized by pairwise conservative, dissipative, and random forces. Acting on particle i by particle j are given by:

$$F_{ij}^{C} = -\alpha_{ij}\omega^{C}(r_{ij})e_{ij} \qquad (1)$$

$$F_{ij}^{D} = -\gamma\omega^{D}(r_{ij})(v_{ij} \cdot e_{ij})e_{ij} \qquad (2)$$

$$F_{ij}^{R} = \sigma\omega^{R}(r_{ij})\xi_{ij}\Delta t^{-1/2}e_{ij} \qquad (3)$$

where $r_{ij} = r_i - r_j$,$r_{ij} = |r_{ij}|$, $v_{ij} = v_i - v_j$ and $e_{ij} = r_{ij}/r_{ij}$, ξ_{ij} is a random number with zero mean and unit variance. For simplicity, the cutoff radius, the particle mass, and the temperature are set to be the units, i.e., $r_c = m = k_B T = 1$. α_{ij} is the repulsion strength which describes the maximum repulsion between interacting particles. ω^C, ω^D, and ω^R are three weight functions for the conservative, dissipative and random forces, respectively. For the conservative force, $\omega^C(r_{ij}) = 1 - r_{ij}/r_c$ for $r_{ij} < r_c$ and $\omega^C(r_{ij}) = 0$ for $r_{ij} \geq r_c$. According to the fluctuation-dissipation theorem, $\omega^D(r_{ij})$ and $\omega^R(r_{ij})$ have a relation,

$$\omega^{D}(r_{ij}) = [\omega^{R}(r_{ij})]^2 \qquad (4)$$

$$\sigma^2 = 2\gamma k_B T \qquad (5)$$

Herein we choose a simple form of ω^D and ω^R due to Groot and Warren,

$$\omega^{D}(r_{ij}) = [\omega^{R}(r_{ij})]^2 = \begin{cases} (1 - r_{ij}/r_c)^2 & (r < r_c) \\ 0 & (r \geq r_c) \end{cases} \qquad (6)$$

And the DPD interaction parameters between particle i and j are given by,

$$\alpha_{ij} = \alpha_{ii} + 3.27\chi_{ij} \quad (\rho = 3)$$

$$\chi_{ij} = \frac{V_{ref}}{RT} * \left[\left(\delta_{Di} - \delta_{Dj}\right)^2 + 0.25 * \left(\delta_{Pi} - \delta_{Pj}\right)^2 + 0.25 * \left(\delta_{Hi} - \delta_{Hj}\right)^2\right] \qquad (7)$$

The DPD interaction parameters between the same and different types of species are α_{ii} (=25) and α_{ij}, respectively. The δ_D, δ_P, δ_H were obtain in handbook, and calculated DPD interaction parameters between different types of beads, α_{ij}, are shown in Table S1.

Table S1. DPD interaction parameters between different beads in the simulations.

Bead type	A	G	P	S
A	25.0			
G	26.3	25.0		
P	25.0	26.3	25.0	
S	32.7	28.0	32.7	25.0

Hansen, C.M. (2007). Hansen Solubility Parameters: A User's Handbook, Second Edition (2nd ed.). CRC Press. https://doi.org/10.1201/9781420006834

As obtained α_{AS} = 32.7 is high enough to lead to the aggregation of monomers before the polymerization, herein a smaller value of α_{AS} = 25.0 is exploited before polymerization, for obtaining the uniform dispersion of the unreacted acrylamide monomers in the solution.

To integrate the Newton's equations of motion, the Groot-Warren-velocity Verlet algorithm is used.

$$r_i(t+\Delta t) = r_i(t) + \Delta t v_i(t) + 1/2(\Delta t)^2 f_i(t)$$
$$\tilde{v}_i(t+\Delta t) = v_i(t) + \lambda \Delta t f_i(t)$$
$$f_i(t+\Delta t) = f_i[r(t+\Delta t), \tilde{v}(t+\Delta t)]$$
$$v_i(t+\Delta t) = v_i(t) + 1/2\Delta t(f_i(t) + f_i(t+\Delta t)). \qquad (8)$$

And $\lambda = 0.65$. In DPD, polymers are constructed by connecting the neighbouring beads together via the harmonic springs $F_i^S = \sum jCr_{ij}$. The spring constant is set to be $C = 10$, and the time step is set to $\Delta t = 0.01$.

2.19.2 Model construction

In the simulations, a three-dimensional simulation box with the size of 50*50*100 in reduced units is constructed, and the periodic boundary conditions are applied in X, Y directions. Virtual walls are settled in the upper and button of simulation box for preventing beads cross over box in Z direction. Based on experimental data, 30,000 A-type monomers, 250 A*-type initiators and 3 layers of graphene oxide consist with G- type are added to the simulation box. The free

space of the box is filled with S-type solvents to represent the solution environment during the diffusion and polymerization process, so that the whole particle number density is kept $\rho = 3.0$; consequently, our DPD model consists of 750,000 movable beads.

A series of DPD simulations is carried out in constant-volume and constant-temperature (NVT) conditions. All simulations are carried out using the GALAMOST package with the SKIPS-GALAMOST reaction simulation platform [S5-S6]. A period of 3×10^5 timesteps simulation is first conducted to relax the configuration before polymerization, followed by 1.2×10^6 timesteps with the polymerization switched on to collect the data and observe the dynamic pathway of polyaniline formation.

2.19.3. Stochastic reaction model for radical polymerization

Since the grafted monomers are locally confined, these grafted monomers in the simulation may be greatly correlated to the mobility of connecting bond. For describing this specific behavior, we use our previously developed stochastic reaction model to control the process. In practice, for any "mobility step", we describe the process as:

$$P^* + A \rightarrow P - A^* \rightarrow P - P^*$$

$$P - P^* + A \rightarrow P - P - P^* \text{ ...(9)}$$

By the stochastic reaction model, assuming that the particle P* with free radical, it will search for all possible particles A within its capture radius. If there are several close A, it firstly chooses one as the object, then another random number is generated. If this random number is smaller than the preset mobility probability P_r, the bond P—A* is generated. In this study, we set the mobility probability as P_r = 0.00005 and the mobility interval $\tau = N_{step}\Delta t = 1$ (i.e., the reaction may take place once within a DPD time unit), which represents a relatively moderate reaction with regard to the simulation time unit according to our previous experiences. Then the A-type bead changes into P-type after polymerization, consequently, a continuous chain mobility process for all monomers will be simulated.

2.20 Statistics Analysis

Each experiment was tested three times in parallel. All experimental results requiring quantitative analysis were analyzed using statistical product and service solution 13.0 (SPSS 13.0) software. Data were expressed as mean ± standard error of the mean.

3 Synthesis

3.1 Synthesis of N-phenyl (4,4′-difluorodiphenyl) ketamine

According to the reported method from previous work of our research group, N-phenyl (4,4′-difluorodiphenyl) ketamine was successfully synthesized.[14,26] The specific synthesis route was shown in **Scheme S1**. 21.82 g (0.10 mol) of 4,4'-difluorobenzophenone, 13.7 mL (0.15 mol) of aniline, 80 mL of toluene and 50.00 g of molecular sieves (4 Å) were added to a 250 ml of three-necked flask equipped with nitrogen vent, mechanical stirring and condensation reflux tube. The reaction was heated to 160 ºC reflux for 24h. Then the reaction was cooled to room temperature. Molecular sieves were filtered, and the obtained filtrate was rotary-evaporated to obtain yellow crystals. The obtained crude product was recrystallized twice with methanol and placed in a vacuum oven at 60 ºC for 24 h. N-phenyl (4,4'-difluorodiphenyl) ketamine was obtained as yellow particles. (23.76 g, 0.08 mol). Yield: 82 %.

3.2 Preparation of Poly (aromatic ether amine) (PEEKt)

According to the previous work in our research group, poly (aromatic ether amine) (PEEKt) was successfully synthesized via **Scheme S2**.[12,27] 6.6066 g (60 mmol) of the 1,4-benzenediol monomer, 15.8388 g (60 mmol) of N-Phenyl (4,4′-difluorodiphenyl) ketamine, 9.9504 g of K_2CO_3, 36.0 mL of toluene, and 60.0 mL of sulfolane were added to a 250 ml of three-necked bottle equipped with mechanical stirring, nitrogen vent and water device. Toluene was added to the three-necked bottle and the water-carrying device for water-carrying. The reaction was raised to 155 ºC for 3h, and toluene was used to reflux with water. The toluene in the reaction was completely released. The reaction was raised to 215 ºC for 6-8h, until the reaction becomes brown and viscous. The reaction solution was poured into 2000ml of ultrapure water, and the product was in the shape of light green thin strips. The product was pulverized into powder by a crusher. At room temperature, the product was stirred and washed 5 times in ultrapure water

for 2 h each time. At the same time, the product was boiled and washed in ethanol for 3 times, 1 h each time. The yellowish solid product was placed in a vacuum oven at 60 ºC for 48 h until the product of PEEKt was completely dry. A yellowish solid was finally obtained. The polymerization yield was as high as 98 %.

3.3 Fabrication of PEEKt membrane

The preparation of PEEKt membrane was carried out by the nonsolvent induce phase separation (NIPS) method. (**Figure S3**) 1.0 g of PEEKt (18 wt%), 0.33 g of PVP (6 wt%), and 4.2 ml of NMP (76 wt%) were placed in a glass lidded vial with stirring and stirred until the solution was brown transparent and viscous. Let the solution stand for 24h until the solution was completely defoamed. The PEEKt membrane was scraped with a 220 μm thick scraper on a glass plate adhered with a non-woven fabric, and the evaporation time was 10 s. The membrane was immediately placed in ultrapure water for 24 h to remove excess solvent and additives. The yellowish PEEKt membrane was obtained.

3.4 Fabrication of PANI@GO/PEEK membrane

PANI@GO/PEEK membranes were prepared by vacuum filtration-assisted deposition and in-situ growth. GO aqueous solutions of different concentrations were prepared. HCl solutions of $(NH_4)_2S_2O_8$ with different concentrations were configurated. The PEEKt membrane was placed in a sand core suction filter unit. 20 ml of GO aqueous solution with the certain concentration was suction filtered to form the brown GO layer on the surface of the PEEKt membrane. 12 ml of $(NH_4)_2S_2O_8$ of HCl solution was poured on the surface of the GO layer, and suctioned for 20 min under vacuum filtration. The surface of the membrane was turned bright yellow immediately. The specific variables are shown in **Tables S2-S4**. $(NH_4)_2S_2O_8$ of HCl solution over the membrane was poured off and let the membrane stand for 10 min. Subsequently, 5 ml of ultrapure water was added on the membrane, and the reaction was allowed to stand for 2 h. The obtained membrane was placed in ultrapure water for 24 h to obtain the black PANI@GO/PEEK membrane.

3.5 Fabrication of GO/PEEKt membrane

GO/PEEKt membranes were prepared by vacuum filtration-assisted deposition. GO aqueous solutions of different concentrations were prepared. The PEEKt membrane was placed in a sand core suction filter unit. 20 ml of 0.5mg of GO aqueous solution was suction filtered to form the brown GO layer on the surface of the PEEKt membrane. The obtained membrane was placed in ultrapure water for 24 h to obtain the black GO/PEEKt membrane.

3.6 Fabrication of PANI/PEEK membrane

4 M HCl solutions of 0.108 M $(NH_4)_2S_2O_8$ were configurated. The PEEKt membrane was placed in a sand core suction filter unit. 12 ml of $(NH_4)_2S_2O_8$ of HCl solution was poured on the surface of the PEEKt membrane under vacuum filtration.[28] The surface of the membrane was turned bright yellow immediately. Let the membrane stand for 10 min. Subsequently, 5 ml of ultrapure water was added on the membrane, and the reaction was allowed to stand for 2 h. The obtained membrane was placed in ultrapure water for 24 h to obtain the navy blue PANI /PEEK membrane.

Scheme S1. Synthesis of N-phenyl (4,4′-difluorodiphenyl) ketimine.

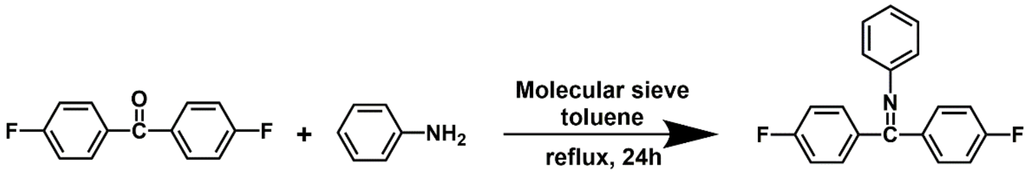

Scheme S2. Synthesis of intermediate polymer PEEKt.

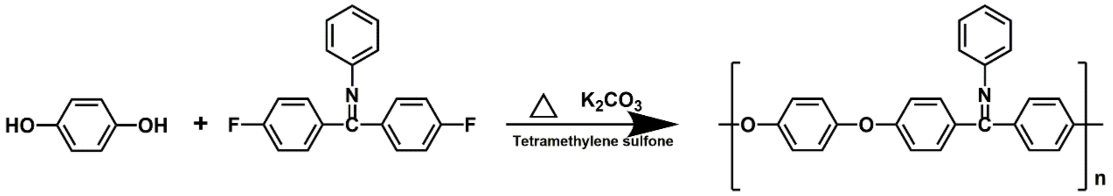

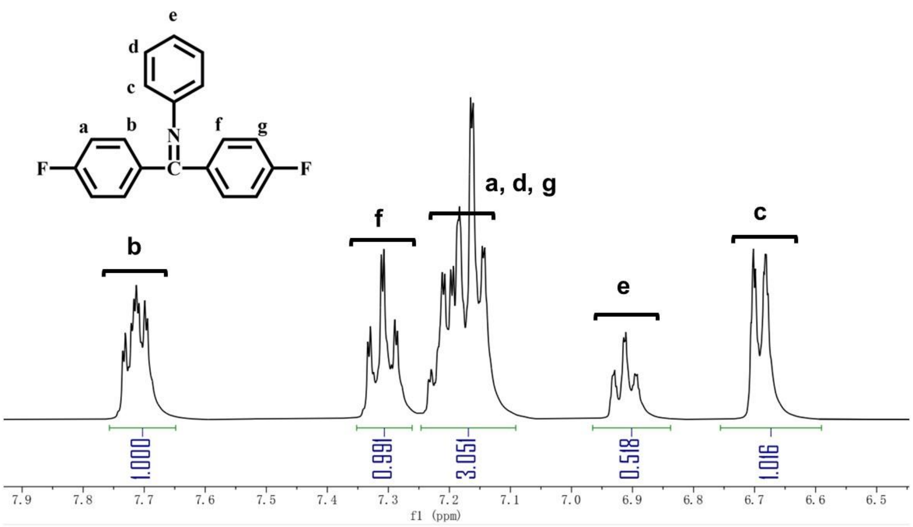

Figure S1. ^{1}H NMR spectrum of N-phenyl(4,4-difluorodiphenyl) ketimine.

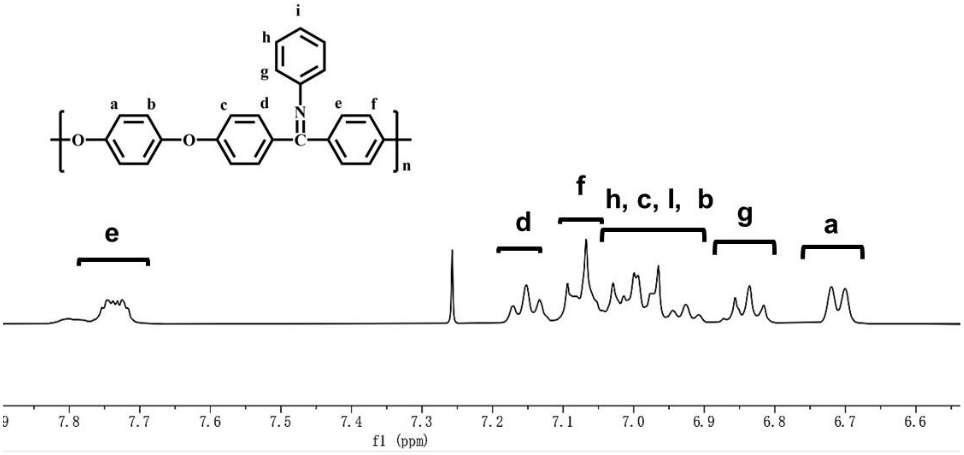

Figure S2. ^{1}H NMR spectrum of PEEKt.

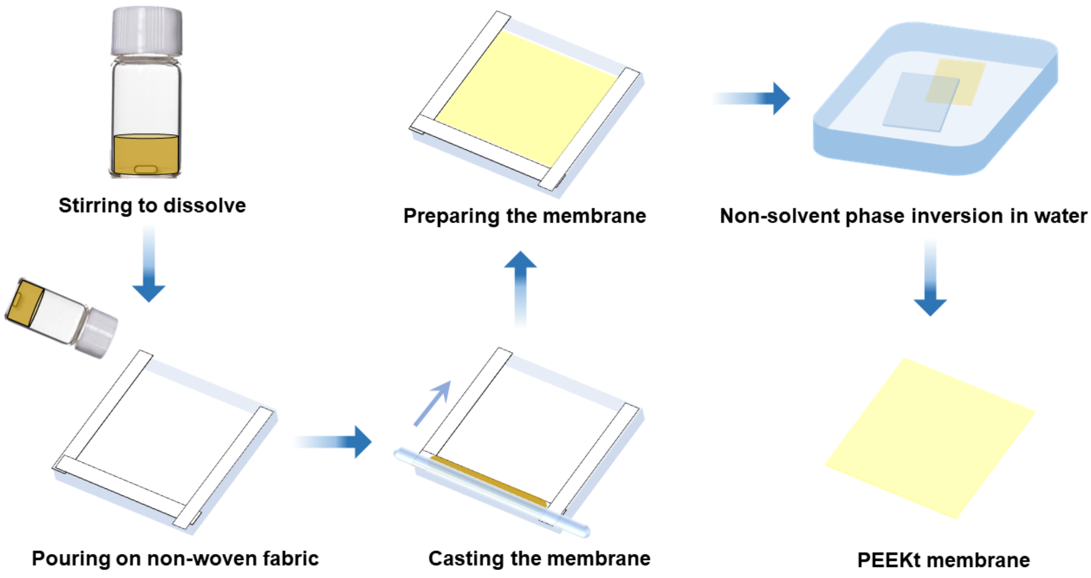

Figure S3. Schematic illustration of the method for PEEKt membranes formation.

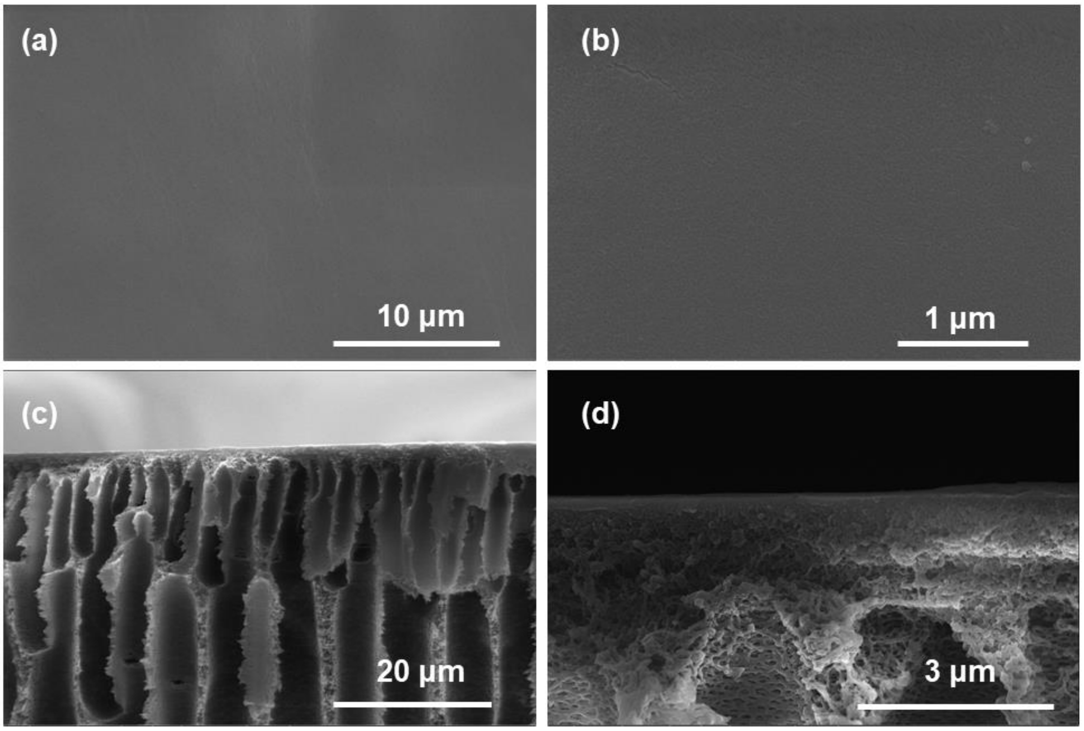

Figure S4. SEM of the PEEKt membranes: (a, b) the top surface SEM images of membranes. (c, d) the cross-sectional SEM images of membranes.

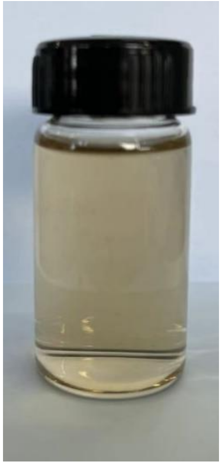

Figure S5. Physical map of dispersion of GO aqueous solution.

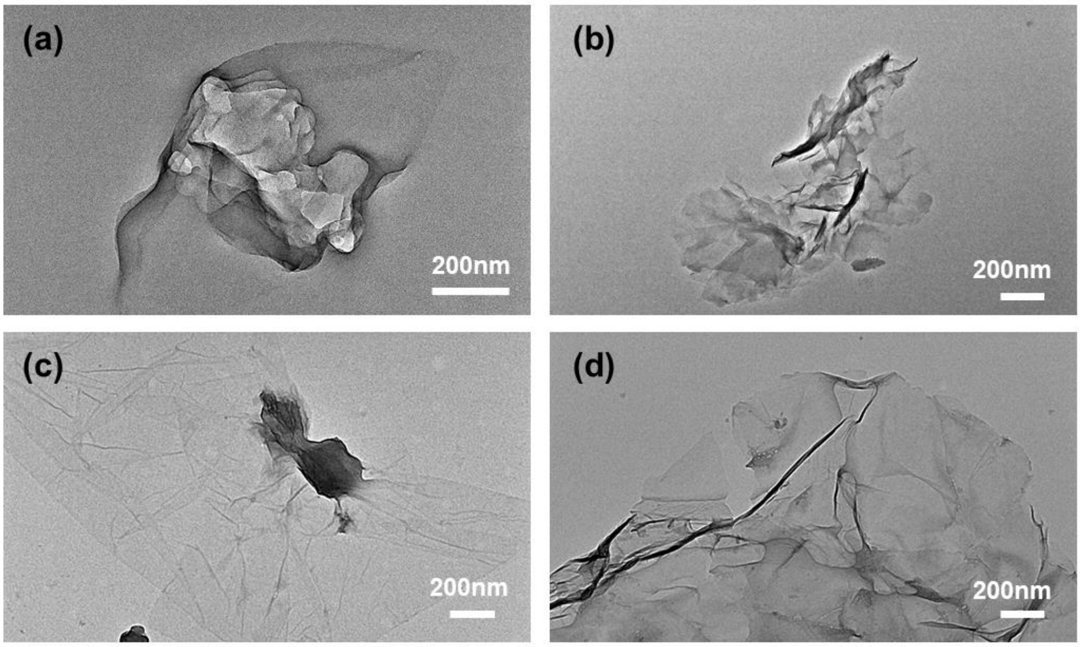

Figure S6. TEM of (a) GO, (b) rGO-NH_2, (c) rGO-COOH and (d) rGO. Scale bar was 200 nm.

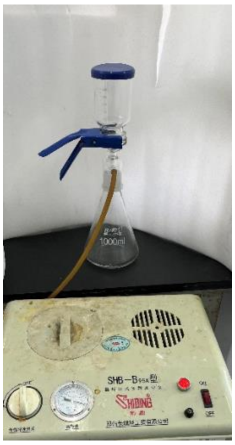

Figure S7. The picture of PANI@GO/PEEK membranes preparation device.

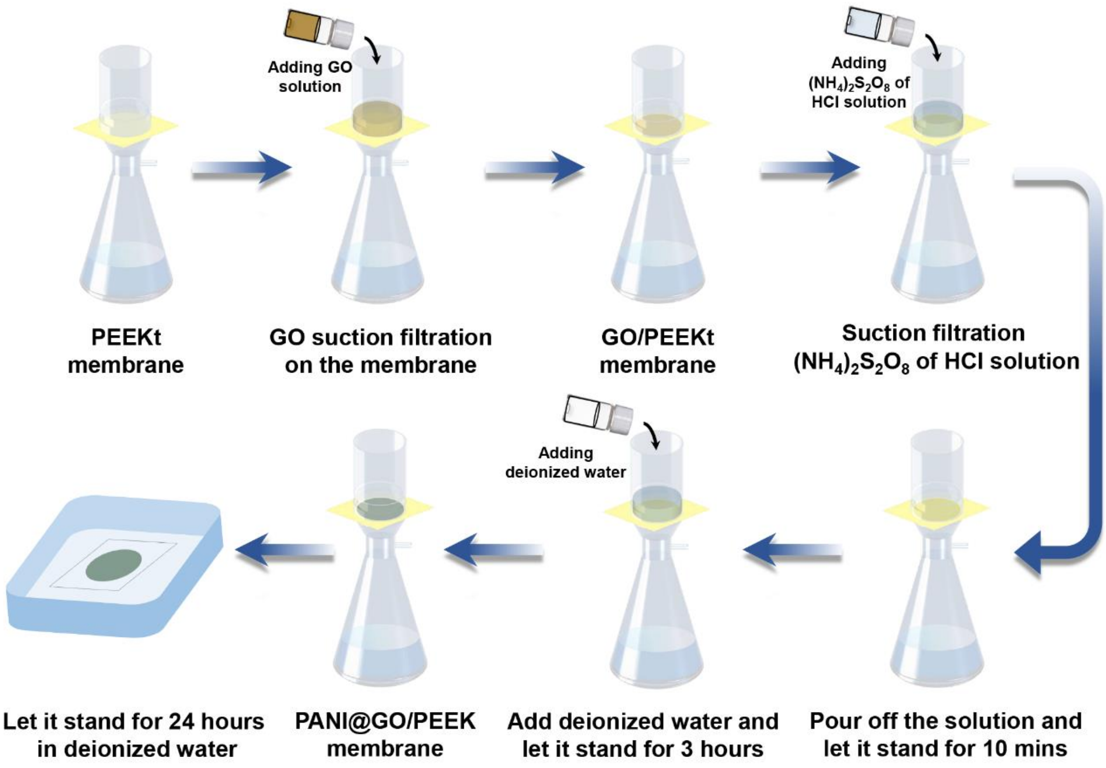

Figure S8. Schematic illustration of the method for PANI@GO/PEEK membranes formation.

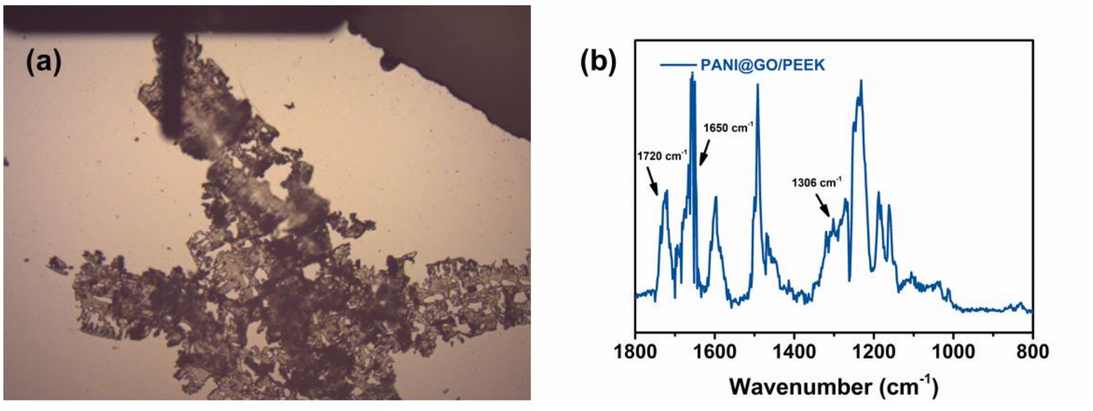

Figure S9. (a) Picture nano-IR of the cross-sectional and (b) nano-IR spectrum of the cross-sectional of PANI@GO/PEEK membrane.

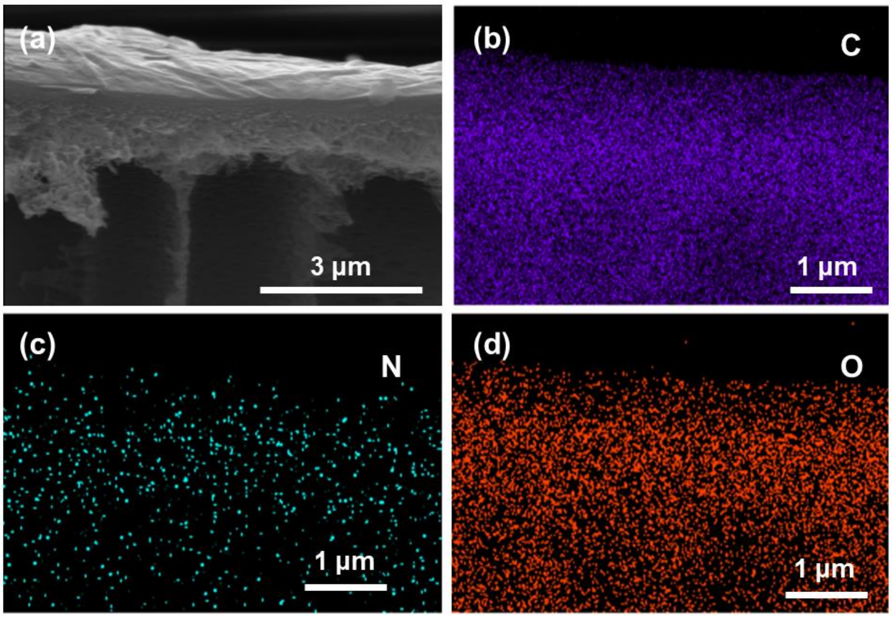

Figure S10. (a) The cross-sectional SEM images of PANI@GO/PEEK membranes. SEM–EDX Kα maps of (b) carbon, (c) nitrogen and (d) oxygen.

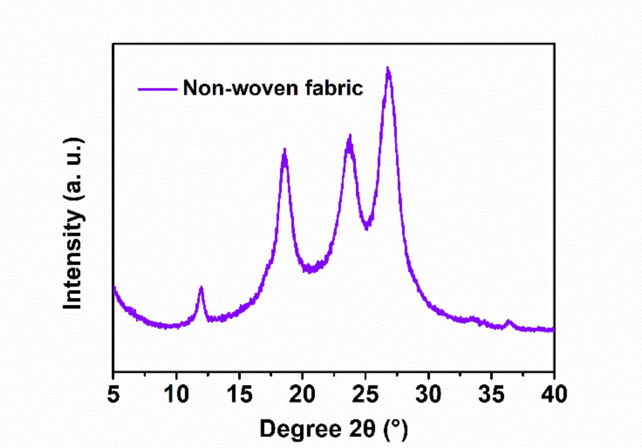

Figure S11. XRD of non-woven fabric.

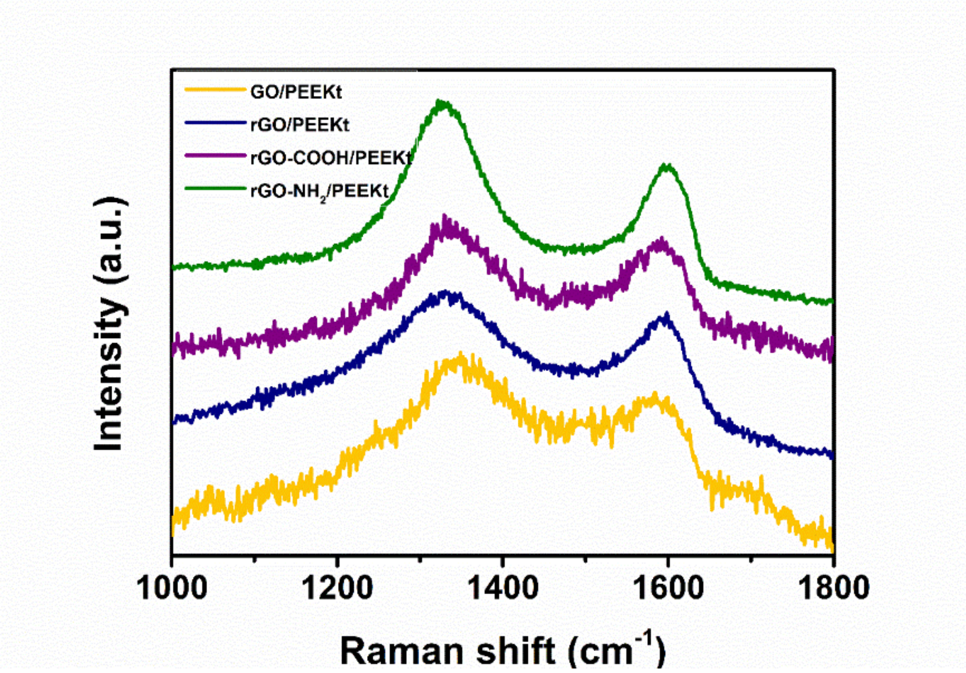

Figure S12. Raman of different membranes.

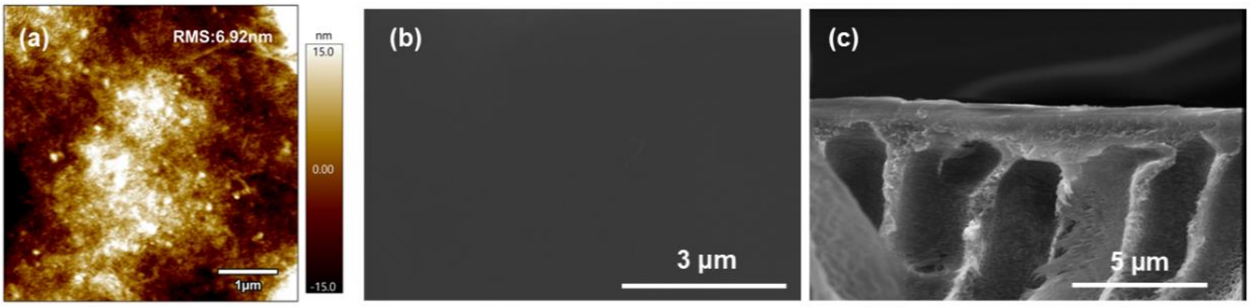

Figure S13. (a) The top surface of AFM, and (b) the top surface and (c) the cross-sectional SEM images of PANI@GO/PEEK membrane.

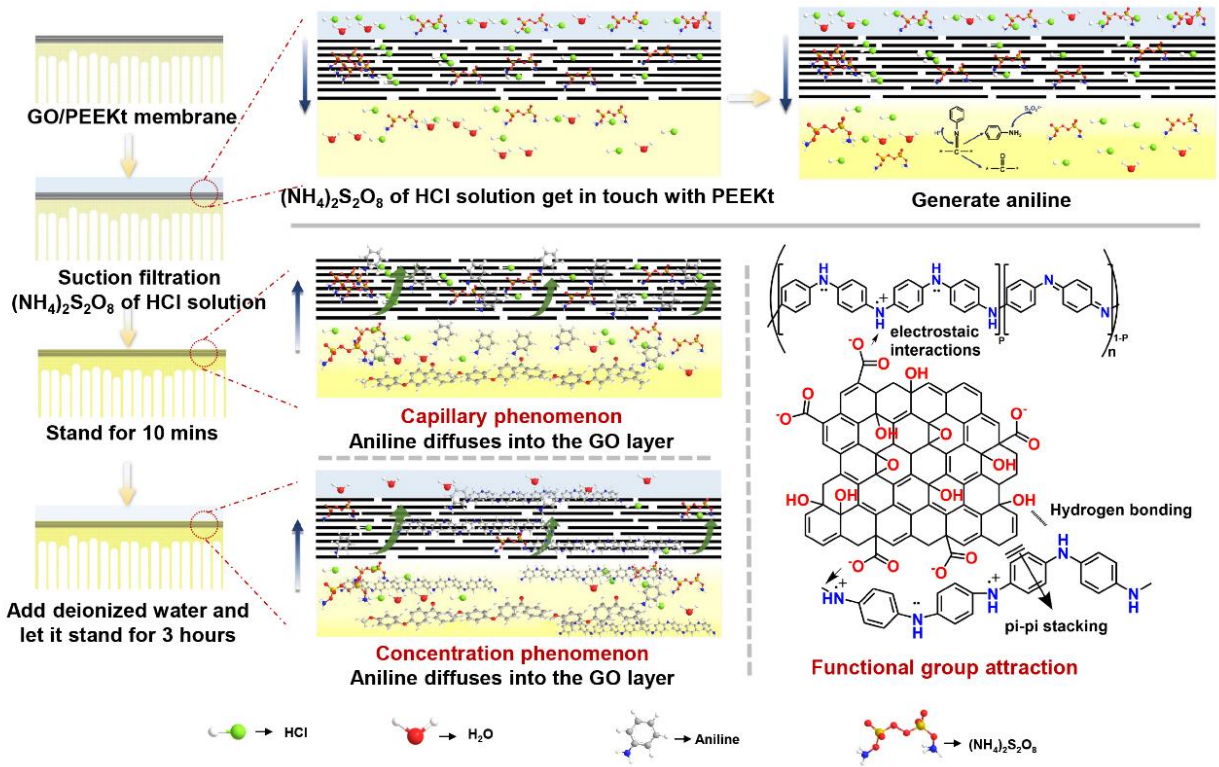

Figure S14. Synthesis mechanism and growth factors of PANI@GO/PEEK membrane.

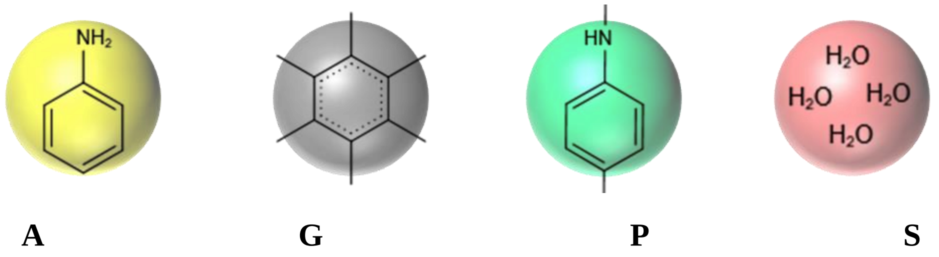

Figure S15. Schematic illustration of the experimentally investigated system and their coarse-grained model.

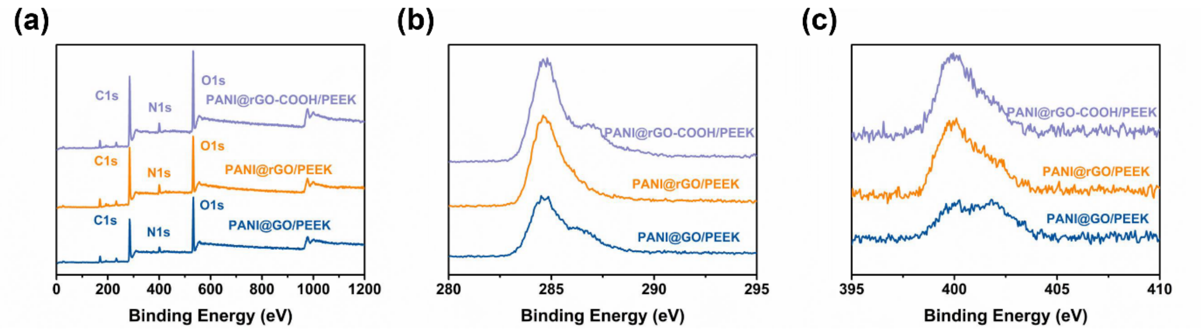

Figure S16. Deconvoluted XPS spectra: (a) The survey spectra of PANI@rGO/PEEK, PANI@rGO-COOH/PEEK and PANI@GO/PEEK membranes; XPS core spectra of (b) C 1s, and (c) N 1s of membranes.

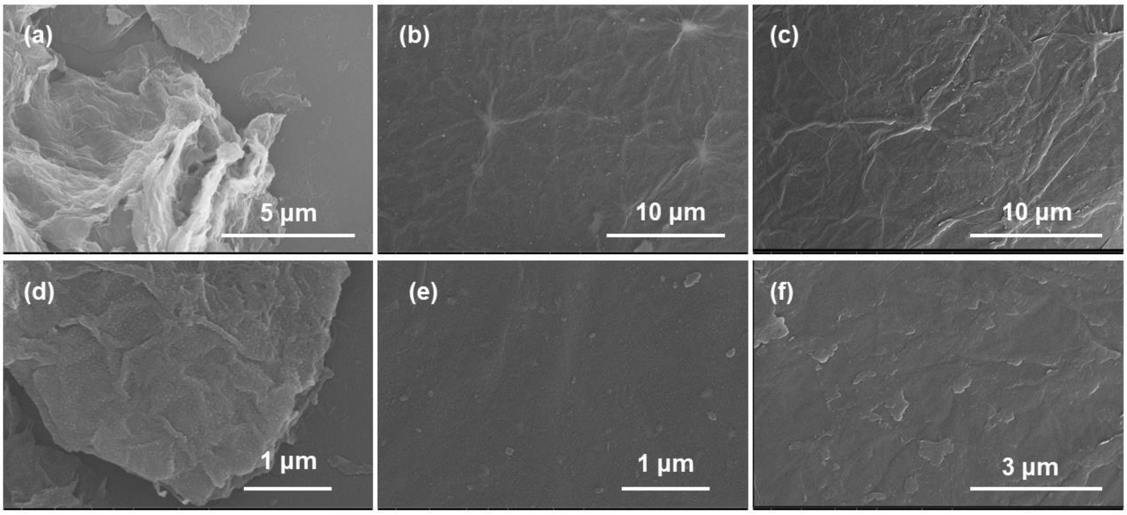

Figure S17. The top surface SEM images of (a, d) PANI@rGO/PEEK membrane, (b, e) PANI@rGO-COOH/PEEK membrane, and (e, f) PANI@GO/PEEK membrane.

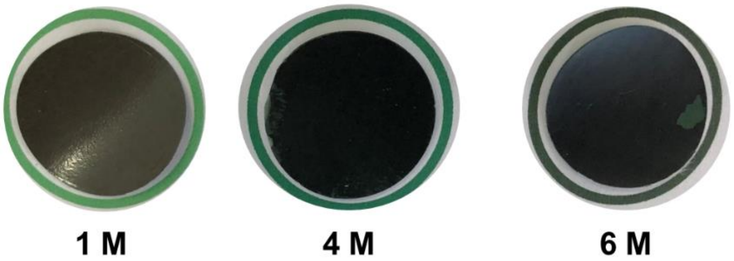

Figure S18. Physical images of PANI@GO/PEEK membrane with different HCl concentration.

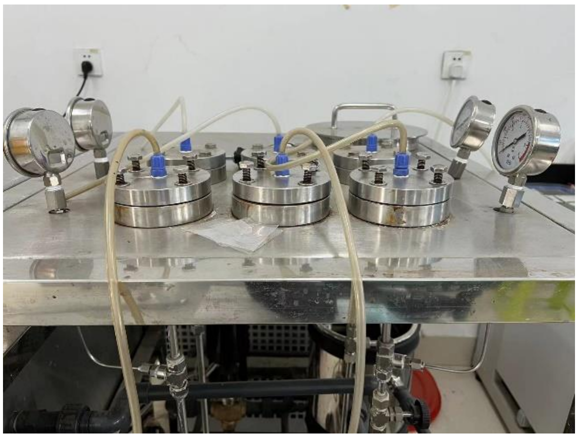

Figure S19. The picture of self-made cross-flow filtration.

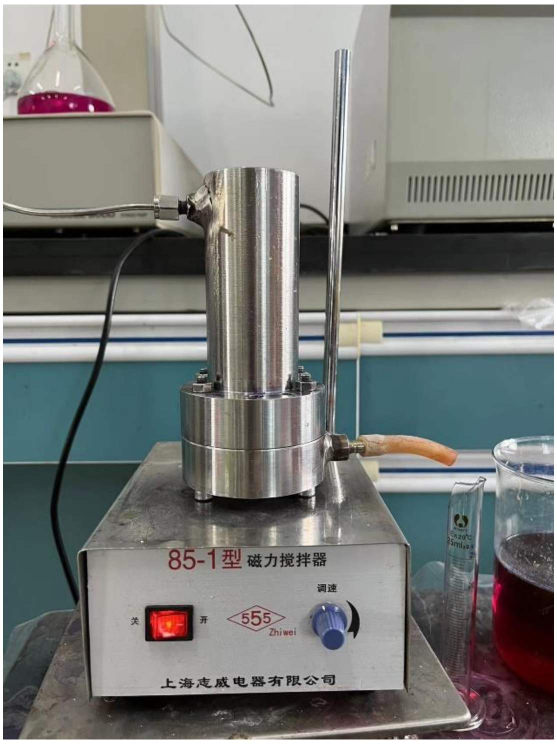

Figure S20. The picture of self-made dead-end filtration.

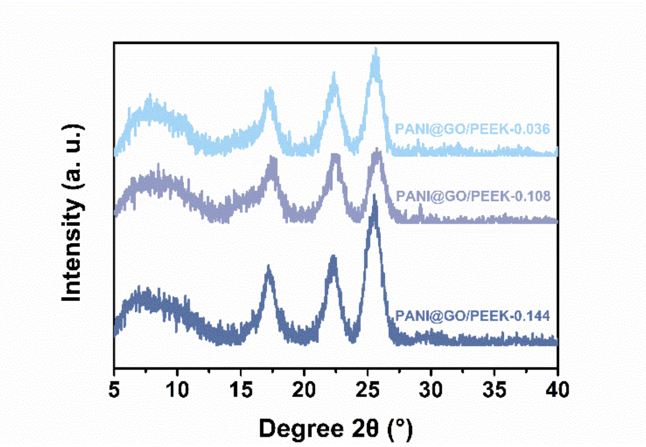

Figure S21. XRD of PANI@GO/PEEK membranes with different treatment c[$(NH_4)_2S_2O_8$] from 0.036 mol/L to 0.144 mol/L.

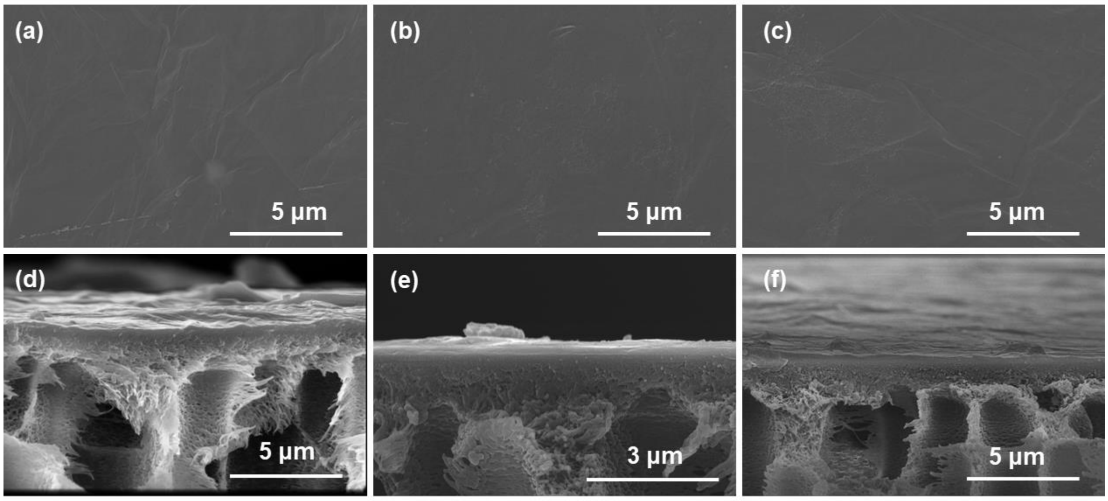

Figure S22. SEM of the as-prepared membranes: PANI@GO/PEEK membranes with different treatment c[$(NH_4)_2S_2O_8$]. (a) 0.036 mol/L, (b) 0.108 mol/L, (c) 0.144 mol/L were the top surface SEM images of membranes. Scale bar was 5 μm; (d) 0.036 mol/L, (e) 0.108 mol/L, (f) 0.144 mol/L were the cross-sectional SEM images of membranes.

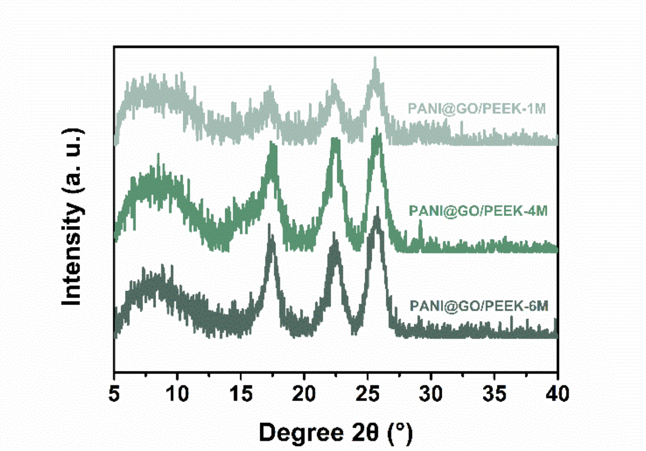

Figure S23. XRD of PANI@GO/PEEK membranes with different treatment c(HCl) from 1.0 mol/L to 6.0 mol/L.

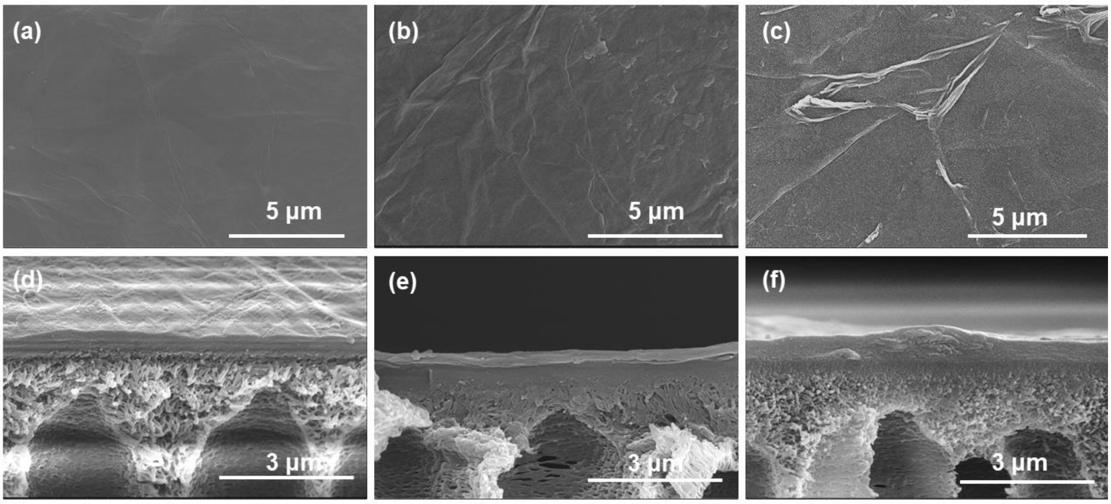

Figure S24. SEM of the as-prepared membranes: PANI@GO/PEEK membranes with different treatment c(HCl). (a) 1.0 mol/L, (b) 4.0 mol/L, (c) 6.0 mol/L were the top surface SEM images of membranes. Scale bar was 5 μm; (d) 1.0 mol/L, (e) 4.0 mol/L, (f) 6.0 mol/L were the cross-sectional SEM images of membranes. Scale bar was 3 μm;

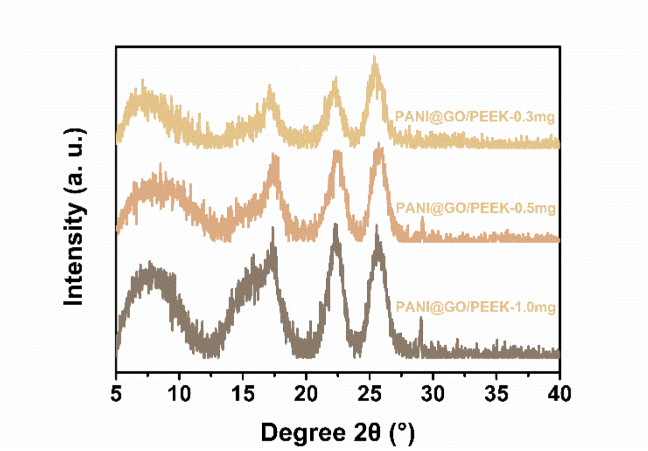

Figure S25. XRD of PANI@GO/PEEK membranes with different treatment m(GO) from 0.3 mg to 1.0 mg.

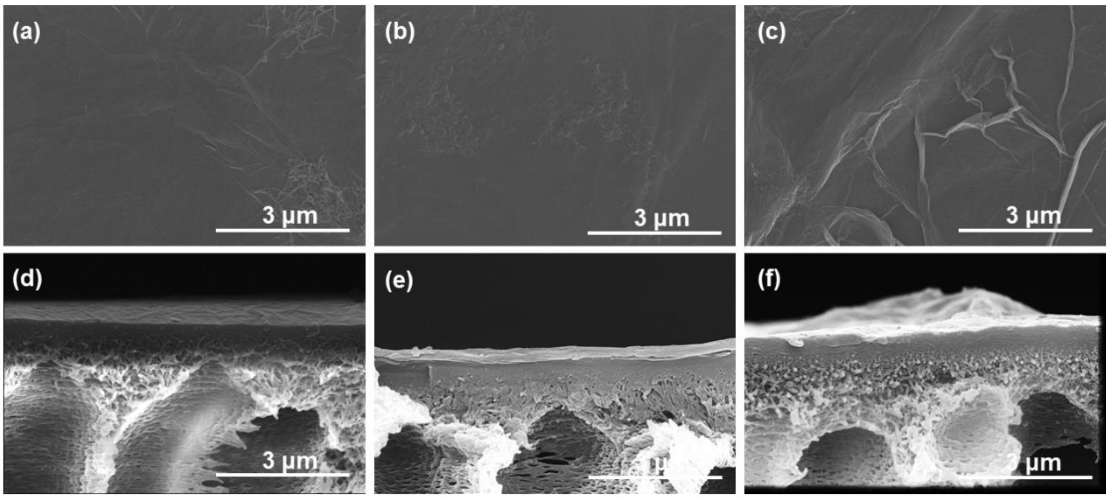

Figure S26. SEM of the as-prepared membranes: PANI@GO/PEEK membranes with different treatment m(GO). (a) 0.3 mg, (b) 0.5 mg, (c) 1.0 mg were the top surface SEM images of membranes. Scale bar was 3 μm; (d) 0.3 mg, (e) 0.5 mg, (f) 1.0 mg were the cross-sectional SEM images of membranes. Scale bar was 3 μm.

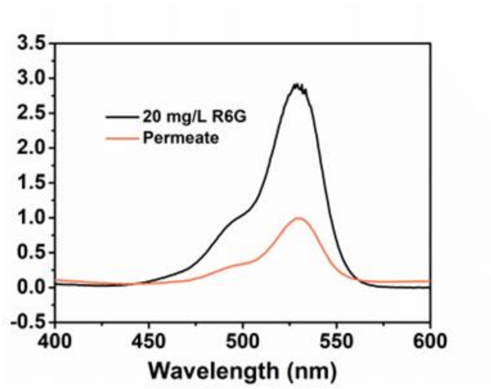

Figure S27. PANI@GO/PEEK membranes UV-Visible spectra of feed dye and permeate.

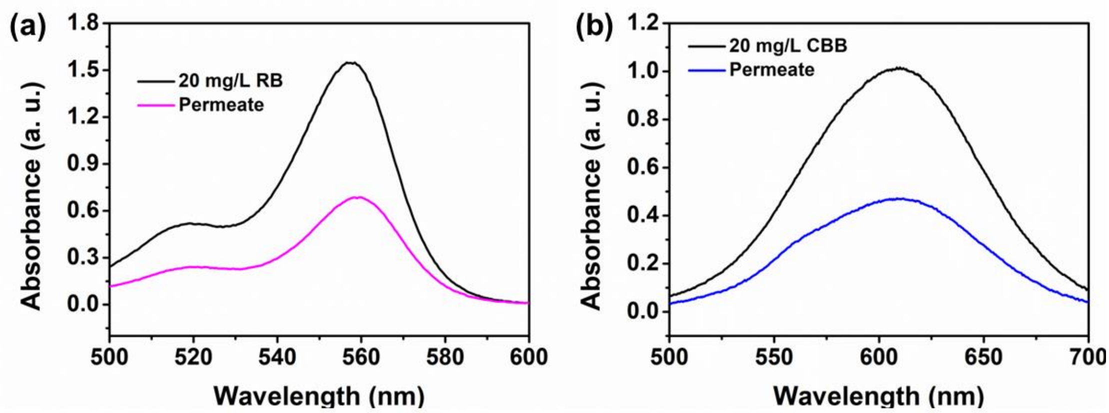

Figure S28. GO/PEEKt membranes UV-Visible spectra of feed dye and permeate.

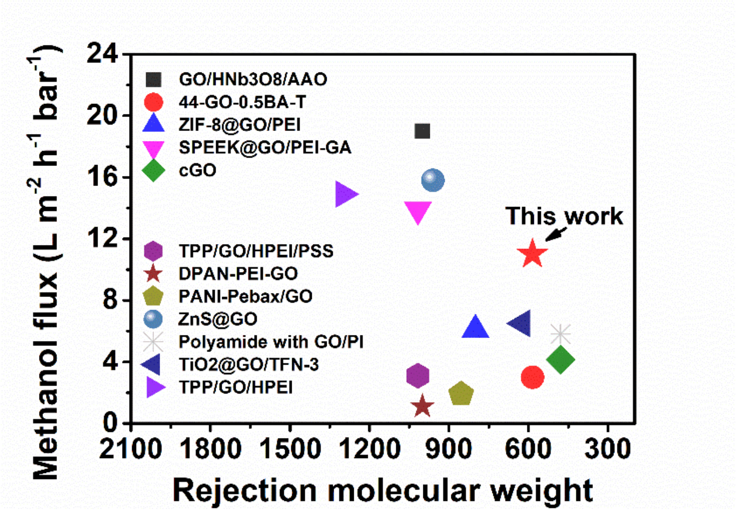

Figure S29. Comparison with others' work. [S7-S17]

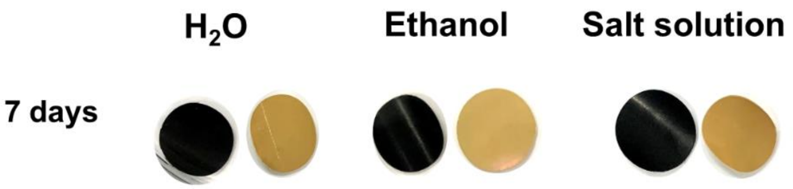

Figure S30. Physical images of GO/PEEKt membrane and PANI@GO/PEEK membrane soaked in water, ethanol, and salt solution.

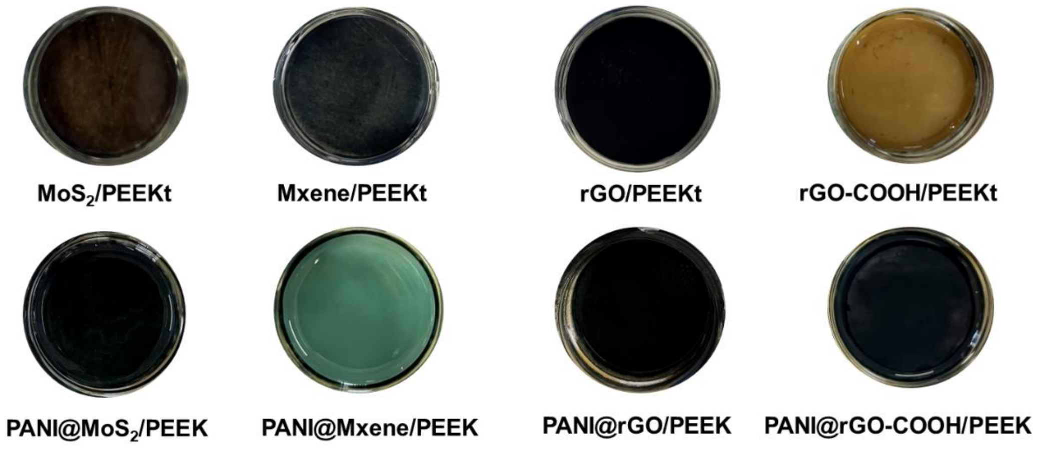

Figure S31. PANI bottom-up growth universal photos of MoS_2, Mxene, rGO and rGO-COOH.

Table S2. PANI@GO/PEEK membrane - $(NH_4)_2S_2O_8$

Sample	$c[(NH_4)_2S_2O_8]$ (mol/L)	c(HCl) (mol/L)	GO (mg/20mL)	Reaction time (h)
PANI@GO/PEEK-0.018	0.018	4	0.5	2
PANI@GO/PEEK-0.036	0.036	4	0.5	2
PANI@GO/PEEK-0.072	0.072	4	0.5	2
PANI@GO/PEEK-0.108	0.108	4	0.5	2
PANI@GO/PEEK-0.144	0.144	4	0.5	2

Table S3. PANI@GO/PEEK membrane - HCl

Sample	$c[(NH_4)_2S_2O_8]$ (mol/L)	c(HCl) (mol/L)	GO (mg/20mL)	Reaction time (h)
PANI@GO/PEEK-1	0.108	1	0.5	2
PANI@GO/PEEK-3	0.108	3	0.5	2
PANI@GO/PEEK-4	0.108	4	0.5	2
PANI@GO/PEEK-5	0.108	5	0.5	2
PANI@GO/PEEK-6	0.108	6	0.5	2

Table S4. PANI@GO/PEEK membrane - m(GO)

Sample	$c[(NH_4)_2S_2O_8]$ (mol/L)	c(HCl) (mol/L)	GO (mg/20mL)	Reaction time (h)
PANI@GO/PEEK-0.2	0.108	4	0.2	2
PANI@GO/PEEK-0.3	0.108	4	0.3	2
PANI@GO/PEEK-0.5	0.108	4	0.5	2
PANI@GO/PEEK-0.75	0.108	4	0.75	2
PANI@GO/PEEK-1.0	0.108	4	1.0	2

Table S5. Information of the dye used in the test.

Dye	Mw(g/mol)	Electric charge	Ultraviolet absorption wavelength (nm)
Rose Bengal	1017	-	548
Coomassie brilliant blue	858.05	-	610
Acid Fuchsin	585	-	546
Rhodamine 6G	479.01	+	554
Methyl Orange	327.33	-	507

Table S6. Comparison of the design and stability of PANI@GO/PEEK membranes with various representative solvent-resistant membranes in the current literature.

**Ref.**	**Material**	**Substrate**	**Substrate Modification**	**Fabrication of Membranes**	**Long-term stability**	**Journal**
19	Perfluoro-alkyl grafted graphene (fGraphene)	Ceramic	Thiol groups -functionalized	Grafted and pressure filtering	Operation up to 80 h	ACS Nano
18	rGO Membrane (UV reduction)	Nylon-6	-	Coating and Drying	Part of the surface is peeled off with tape	ACS Nano
20	COO⁻−GO@PILTf2N−AT	Nylon-66	-	Vacuum-assisted Filtration	Operation up to 150 h	ACS Nano
38	grafted PNIPAM onto GO (PGOMs)	Cellulose Acetate	-	Vacuum-assisted Filtration	-	Advanced Functional materials
22	MXene/BN@PDA/PEI membrane (covalent cross-linking)	PVDF	-	Vacuum-assisted Filtration	Immersed for 600 h	Advanced Functional materials
23	ACGMs (carbonized chitosan (CS) into thermally reduced GO nanosheets)	Aluminum Oxide substrates	-	Vacuum-assisted Filtration	Operation up to 80 h	Advanced materials
39	MoS2-amide<br>(MoS_2 grafted with 2-iodoacetamide)	Nylon	-	Vacuum-assisted Filtration	Immersed for 14 days	Advanced materials
40	MXene@PSS<br>(PSS introduced into the laminar stack of titanium carbides of MXene)	PES	-	Vacuum-assisted Filtration	Operation up to 50 h	Angewandte Chemie

15	CP5-MXene (MXene nanosheets with pillararenes CP5)	PES	-	Vacuum-assisted Filtration	Operation up to 192 h	Angewandte Chemie
16	GO–PA (The ionic channels played by polyamine macromolecules (PA) and GO)	Anodisc 47 and PES	-	Vacuum-assisted Filtration	Operation up to 20 h	Nature Nanotechnology
42	nanoporous MoS2 NSs (a mixture of one-to-two-layer-thick porous NSs and nanodisks (NDs).)	Anodisc alumina	-	Vacuum-assisted Filtration	Operation up to 160 h	Nature Communications
43	Al^{3+}-intercalated MXene membranes	PES	-	Vacuum-assisted Filtration	Operation up to 400 h (U-shaped device)	Nature Sustainability
17	GO-TBO (polycyclic dye toluidine blue O, π-conjugated polycyclic cations)	PES	-	Vacuum-assisted Filtration	Operation up to 700 h	Nature Sustainability
4	SFGO-La^{3+} and LFGO-La^{3+} membranes	Nylon	-	Vacuum-assisted Filtration	Operation up to 25 h (cross-flow)	Science Advances
41	functionalized graphene oxide membranes (FGOMs) with nitrogen groups such as amine groups and polarized nitrogen atoms	Aluminum Oxide substrates	-	Vacuum-assisted Filtration	Operation up to 35 h	Journal of the American Chemical Society
24	GO/ ZIF-8 hybrid membrane	Ceramic tube	PDA-modified	Vacuum-assisted filtration and In-situ growth	Operation up to 180 h	Nature Nanotechnology
-	**PANI@GO/PEEK membrane**	**PEEK**	**PANI modification of PEEK and GO**	**Vacuum-assisted filtration and in-situ integrated growth**	**Immersed for 240 days Operation up to 100 h**	**This work**

References

[S1] E. G. Flekkoy, P. V. Coveney, G. De *Physical Review E* **2000**, 62 (2), 2140-2157.

[S2] H. Liu, M. Li, Z.-Y. Lu, *Macromolecules* **2009**, 42 (7), 2863-2872.

[S3]H. Liu, M. Li, Z.-Y. Lu, *Macromolecules* **2011**, 44 (21), 8650-8660.

[S4] H. Liu, H. J. Qian, Y. Zhao, *Journal of Chemical Physics* **2007**, 127 (14), 144903.

[S5] H. Liu, Y. L. Zhu, Z. Y. Lu *J. Comput. Chem.* **2016**, 37 (30), 2634-2646.

[S6] Y. L. Zhu, H. Liu, Z. W. Li, *J. Comput. Chem.* **2013**, 34 (25), 2197-211.

[S7] T. Gao, H. Wu, L. Tao, *J. Mater. Chem. A* **2018**, 6,19563.

[S8] K. Nakagawa, M. Kunimatsu, K. Yasui, *J. Membr. Sci.* **2021**, 640, 119799.

[S9] H. Yang, N. Wang, L. Wang, *J. Membr. Sci.* **2018**, 545, 158-166.

[S10] N. Cao, Z. Lin, R. Sun, *Carbon* **2021**,185, 39e47.

[S11] Y. Li, C. Li, S. Li, *J. Mater. Chem. A* **2019**, 7,13315.

[S12] H. Abadikhah, E. N. Kalali, S. Behzadi, *Chem. Eng. J.* **2019**, 204, 99-109.

[S13] D. Hua, T. S. Chung, *Carbon* **2017**,122, 604-613.

[S14] R. Ding, H. Zhang, Y. Li, *Chem. Eng. Sci.* **2015**, 138, 227-238.

[S15] J. Aburabie, K. V. Peinemann, *J. Membr. Sci.* **2017**, 523, 264-272.

[S16] N. Wang, H. Sun, H. Yang, *ACS Appl. Nano Mater.* **2020**, 3, 5874−5880.

[S17] J. Su, X. Lv, S. Li, *Separation and Purification Technology* **2022**, 278, 119567.
